# Abstracts from the 2nd International Conference of Science, Technology, Education, and Management (InSTEM 2023)

**DOI:** 10.1186/s12919-024-00294-1

**Published:** 2024-06-06

## O1 Instagram usage pattern and social comparison as the predictors of body dissatisfaction: a study on Malaysian university students

### Bain Lok Peang Yee, Law Mei Yui

#### Tunku Abdul Rahman University of Management and Technology, Kuala Lumpur, Malaysia

##### **Correspondence:** Law Mei Yui (lawmy@tarc.edu.my)

*BMC Proceedings 2024*, **18(9):**O1

**Background**: Instagram is the most popular platform full of aesthetic and beautified posts that most university students are fascinated with. This has in turn raised a concern on body image among university students. The primary objective is to identify Instagram usage patterns among university students and assess whether these patterns predict body dissatisfaction and social comparison in predicting body dissatisfaction.

**Materials and methods:** A cross-sectional quantitative research design was adopted to identify the Instagram usage patterns of university students. Using a purposive sampling method, 117 university students from private institutions, all falling within the age range of 18 to 26 were involved. To gather data, various questionnaires were administered through Google Forms. These included the Adapted Passive Active Use Measure, Adapted Iowa-Netherlands Comparison Orientation Measure, and Body Shape Questionnaire-8C. SPSS version 26 was utilized to analyse the data collected.

**Results:** The findings from the descriptive analysis revealed that 25.60% of the participants were active Instagram users, while 74.40% of participants were passive Instagram users. It shows most university students were passive Instagram users. Results from simple linear regression analysis indicated that both active [β =.55, t (115) = 3.45, *p* <.05] and passive [β =.59, t (115) =3.99, *p* <.05] Instagram usage patterns have significantly predicted body dissatisfaction. Besides, the result from simple linear regression analysis indicated that social comparison [β =.27, t (115) = 2.19, *p* <.05.] significantly predicted body dissatisfaction.

**Conclusion:** This research underscores the importance of considering Instagram usage patterns and the impact of social comparison in the context of body dissatisfaction experienced by university students. Several authorities should be concerned about the factor of Instagram usage patterns to body dissatisfaction among university students. Awareness of the negative effects of Instagram usage patterns and social comparison should be brought up among university students.

## O2 A retrospective study on ventricular septal defect patients with pulmonary arterial hypertension: a single centre study

### Prema Subramaniam^1,2^, Vasudevan Ramachandran^1^, Harris Shah Abdul Hamid^3^, Geetha Kandavello^2^

#### ^1^Department of Medical Sciences, Faculty of Health Sciences, University College of MAIWP International, Kuala Lumpur, Malaysia; ^2^Department of Paediatric, National Heart Institute, Kuala Lumpur, Malaysia; ^3^Department of Education and Psychology, Faculty of Management, Education, and Humanities, Universiti College of MAIWP International, Kuala Lumpur, Malaysia

##### **Correspondence:** Vasudevan Ramachandran (drvasu@ucmi.edu.my)

*BMC Proceedings 2024*, **18(9):**O2

**Background**: The purpose of this study was to evaluate the demographic and clinical characteristics of the Ventricular Septal Defect (VSD) subjects with pulmonary arterial hypertension (PAH) from a single centre registry. PAH is a pathophysiological disorder that may involve multiple clinical conditions and may be with a variety of cardiovascular diseases. Furthermore, VSD was the most frequent underlying defect among PAH patients.

**Materials and methods**: A retrospective analysis of 31 VSD patients with PAH was recorded from November 1992 to February 2023 admitted at the National Heart Institute (NHI) Cardiology Unit in Kuala Lumpur, Malaysia.

**Results**: Of the 31 VSD patients with PAH, 54.8 % were female and 45.2% of subjects were male. The age group from 10-19 years shows the highest (38.7%) number of patients compared to other age groups. Out of the nine states recorded, patients from Selangor state showed the highest (25.8%) number of patients admitted at NHI. Among all the three ethnics, Malay was higher (54.8%) followed by Indian (22.6%) and Chinese (22.6%) ethnics, respectively. The management therapy received by the patients is mostly monotherapy (45.2%), dual therapy (32.3%) and followed by triple therapy (6.5%). Cardiac catheterization was performed for all the 31 patients. Serum pro-brain natriuretic peptide (NTproBNP) biomarker was considered as a risk factor for VSD with PAH (522.32 ±751.496), mean pulmonary artery pressure (mPAP) (68.77±19.502) mean arterial pressure (91.16±13.337), and pulmonary flow/systemic flow; (Qp/Qs) (1.5± 0.580) were higher than the normal values. The most common monotherapy used was sildenafil (58.1%), followed by bosentan (32.2%).

**Conclusion**: Identification of factors that affect prognosis, in particular, is crucial for preventing rapid progression. The NTproBNP, mPAP, mean arterial pressure and pulmonary flow/systemic flow play an important role in the development of VSD with PAH. Better strategies to improve awareness, screening, treatment, and management of VSD with PAH are required.

## O3 Functional MRI-based brain structure and neuronal connectivity of default mode network in Alzheimer’s Disease and healthy controls correlated with *APOE* ε4 carrier status

### Mohad Azmi Nur Hafizah^1^, Ibrahim Nur Shahidatul Nabila^1^, Ibrahim Buhari^1^, Seriramulu Vengkatha Priya^1^, Mohamad Amin Amrina^4^, Karuppiah Thilakavathy^5^, Mohamad Mazlyfarin^8^, Omar Nur Farhayu^1^, Abu Hassan Hasyma^1^, Ibrahim Normala^6^, Razali Rizah Mazzuin^9^, Harrun Noor Harzana^10^, Sallehuddin Hakimah^7^, Suppiah Subapriya^1,2,3^

#### ^1^Radiology Department, Faculty of Medicine and Health Sciences, Universiti Putra Malaysia, Selangor, Malaysia; ^2^Center for Nuclear Diagnostic Imaging, Universiti Putra Malaysia, Serdang, Selangor, Malaysia; ^3^Nuclear Imaging Unit, Hospital Sultan Abdul Aziz Shah, Selangor, Malaysia; ^4,5,6,7^Faculty of Medicine and Health Sciences, Universiti Putra Malaysia, Selangor, Malaysia; ^8^Imaging Diagnostic and Radiotherapy Department, Faculty of Allied Health, Universiti Kebangsaan Malaysia, Jalan Raja Muda Abdul Aziz, Kuala Lumpur, Malaysia; ^9^Department of Medicine, Hospital Kuala Lumpur, Jalan Pahang, Kuala Lumpur, Malaysia; ^10^Family Medicine Unit, Klinik Kesihatan Pandamaran, Persiaran Raja Muda Musa, Klang, Malaysia

##### **Correspondence:** Suppiah Subapriya (subapriya@upm.edu.my)

*BMC Proceedings 2024*, **18(9):**O3

**Background**: Late-onset Alzheimer’s disease (LOAD) is caused by *APOE* gene mutations. *APOE* ε4 allele is linked to increased vulnerability for the development of LOAD. *APOE* genotyping and multiparametric resting state functional MRI (rs-fMRI) tests for LOAD may help to better characterise the disease. The main aim of this study is to analyse and compare structural and functional connectivity seen on MRI with *APOE* ε4 carrier status in LOAD and healthy control (HC) subjects.

**Materials and methods:** In this study, clinicians’ assessment, MOCA, MMSE, and CDR scores were utilized to categorise subjects as LOAD or HC. We performed rs-fMRI on 15 LOAD (aged 65–83 years) and 15 HC (aged 60–82 years). The structural voxel-based morphometry (VBM) model was used to process participants’ MRI data sets’ regional anatomy. The CONN Toolbox utilized seed-based methodology to examine the functional MRI dataset. DNA genotyping was performed on three LOAD and three HC subjects. RE *AFIII* targeted rs429358 and *HAEII* targeted rs7412 in PCR-RFLP genotyping was performed.

**Results:** Overall LOAD had lower brain volume and VBM activation than HC. Seed-based rs-fMRI functional connectivity in the DMN nodes showed larger spatial activation sizes but decreased activation intensity in LOAD compared to HC. Three individuals were identified with a homozygous *APOE* ε4 negative genotype (non-carriers), which aligns with the HC genotype, whereas individuals with a heterozygous genotype positive for *APOE* ε4, were well matched with the structural and rs-fMRI features consistent with LOAD.

**Conclusion:** The neuroimaging MRI findings related to the brain structure and functional connectivity were well matched with the *APOE* ε4 carrier status in individuals with LOAD compared with HC subjects. Genotyping coupled with radiomics has the potential to enhance the characterisation and prognostication and contribute to better management of Alzheimer’s disease.

## O4 Effects of underfilled EDTA vacutainer on automated blood cell indices and microscopic examination in dengue diagnosis

### Sarifah Ahiriel^1,2^, Hamidah Abu Bakar^1,3^

#### ^1^Health Science Department, Faculty of Engineering and Life Sciences, Universiti Selangor (UNISEL), Shah Alam, Selangor, Malaysia; ²Haematology Section, Pathology Unit, Hospital Tuaran, Tuaran, Sabah; ^3^Faculty of Pharmacy, University College of MAIWP International, Kuala Lumpur, Malaysia

##### **Correspondence:** Hamidah Abu Bakar (dr.hamidah@ucmi.edu.my)

*BMC Proceedings 2024*, **18(9):**O4

**Background:** Dengue incidence has increased dramatically around the world in recent decades and requires quick and proper intervention. Dengue infection is diagnosed by various serology tests to confirm the diagnosis. However, these serological tests may not be available in some small hospitals or clinics. A full blood count is a basic routine test for detecting dengue infection before proceeding to confirmation tests. Nevertheless, samples are often received underfilled, which is the main reason for sample rejection as they do not comply with the standard guidelines and lead to unnecessary sample rejection. This could increase the workload of health practitioners, waste time and materials, and delay treatment. This study investigated the effect of underfilled ethylenediaminetetraacetic acid (EDTA) vacutainer on the automation of red blood cell indices and morphology.

**Materials and methods:** 121 patients from Hospital Tuaran, Sabah, participated in this study. Each patient had 7.0 to 8.0 ml of venous blood drawn and transferred into four 3.0 ml EDTA vacutainers filled with different volumes: 3.0 ml (standard volume according to the Clinical Laboratory Standards Institute), 2.5 ml, 1.5 ml, and 0.5 ml. The One-Way ANOVA was employed to calculate the mean difference in blood cell indices for gender while Chi-square was used to calculate the frequency of RBC morphology and PLT clumping that showed in percentage.

**Results:** Underfilling EDTA did not affect red blood cell indices or platelet clumping in either males or females. However, under filling EDTA had a significant effect on red blood cell morphology (*p*<0.05).

**Conclusion:** Under the circumstances of the present study, present data appear that under filled EDTA vacutainer samples do not affect blood cell indices, but they may affect microscopic examination. While it is recommended to follow the standard guidelines, however, under filled EDTA vacutainer outcomes should be considered especially if the dengue tests are not well accessible.

## O5 Unlocking the e-wallet craze: examining students’ intentions to use e-wallets in higher learning institutions in the Klang Valley

### Zuraini Alias, Norhazwani Mohamed Asri, Siti Fatinnah Ab Rahman, Alvien Haniza Rahman Ajip, Nuralia Syafiqa Amran, Amani Anas, Afif Aniq Jasni

#### Department of Management, Faculty of Management, Education, and Humanities, University College of MAIWP International, Kuala Lumpur, Malaysia

##### **Correspondence:** Zuraini Alias (drzuraini@ucmi.edu.my)

*BMC Proceedings 2024*, **18(9):**O5

**Background**: The rapid advancement of technology has led to changes in the way we handle payments. Among these technological developments, the electronic wallet (e-wallet) has emerged. E-wallets play a role in simplifying daily transactions for users, enabling them to conduct financial activities without relying on physical cash. Notably, e-wallets also contribute to reducing the risk associated with carrying physical currency, making them a more secure option. The advantages of e-wallets are indeed noteworthy. Thus, this study aimed to investigate the factors influencing individuals’ willingness to adopt e-wallets, with a particular focus on students in higher education institutions.

**Materials and methods:** An online survey using Google Forms was administered to 153 respondents enrolled in higher education institutions within the Klang Valley area. The collected data underwent both descriptive and inferential statistical analyses, employing methods such as Pearson Correlation and Multiple Linear Regression. Furthermore, Cronbach’s α coefficient was employed to assess the internal consistency reliability of the data using the Statistical Package for the Social Sciences (SPSS).

**Results:** The findings of the study revealed that three key factors significantly influence the intention to use e-wallets: Perceived Usefulness (PU), Perceived Ease of Use (PEoU), and Perceived Risk (PR). It is important to note, however, that the study also uncovered that higher levels of perceived risk might impede users from adopting e-wallets. These findings are valuable for e-wallet service providers, as they aid in recognizing the pivotal factors that influence users’ intentions to embrace e-wallet services.

**Conclusion:** The results of our analysis revealed a significant association between the intention to use e-wallets and the factors of perceived usefulness, perceived ease of use, and perceived risk. This assertion is supported by the p-values obtained through Pearson’s correlation analysis, all of which surpassed the conventional threshold of 0.05.

## O6 Assessing ICT proficiency and competence among Islamic kindergarten teachers for online teaching and learning

### Siti Syazwani Zulkuple, Mohd Shawani Ahmad Sabri, Saleha Idris

#### Department of Education and Psychology, Faculty of Management, Education, and Humanities, University College of MAIWP International, Kuala Lumpur, Malaysia

##### **Correspondence:** Siti Syazwani Zulkuple (syazwani@ucmi.edu.my)

*BMC Proceedings 2024*, **18(9):**O6

**Background**: This study aims to identify the level of ICT knowledge and skills of Islamic Kindergarten teachers in handling ICT for online teaching and learning.

**Materials and methods:** A quantitative approach was used in this study. The selection of respondents was carried out by purposive sampling, involving 110 teachers who teach at Tadika Islam (MAIWP). A questionnaire regarding the level of expertise and competence of preschool teachers in integrating information and communication technology (ICT) in the classroom was used as a research instrument. Research data were analysed using descriptive analysis and inferential analysis, including Pearson’s correlation and t-tests.

**Results:** The findings show that the majority of 62 teachers (56.4%) have a moderate level of ICT knowledge (mean=2.36, sp= 0.554). Additionally, the level of ICT skills of teachers in handling ICT for online teaching and learning was found to be at a high level (64.5%, mean=2.64, sp= 0.480). The Pearson correlation analysis test revealed a significant relationship between teachers’ ICT knowledge and teachers’ competence in handling ICT for online teaching and learning. Furthermore, a significant relationship was observed between teachers’ ICT skills and teachers’ competence in this regard. However, the t-test analysis indicated no significant difference in the level of ICT knowledge based on teachers’ period of teaching experience. Nevertheless, there was a significant difference in teachers’ ICT skills based on their period of teaching experience.

**Conclusion:** In conclusion, this study highlights the need for ICT training and workshops for teachers to enhance their ICT knowledge and skills. The provision of technological materials in kindergartens is also essential to facilitate the application of ICT knowledge and the development of teachers’ skills in using ICT for teaching and learning.

## O7 Psychometric analysis of a compulsory college-level English communication course examination paper

### Harris Shah Abd Hamid^1^, Khairul Firdaus Ne’matullah^2^_,_ Adiba Zailan^2^, Khadijah Ummira Muhammad Helmi^2^, Fazlina Ilyani Kamaruddin^2^, Mohd Azhar Kamarudin^2^

#### ^1^Department of Education and Psychology, Faculty of Management, Education, and Humanities, Universiti College of MAIWP International, Kuala Lumpur, Malaysia; ^2^Department of Languages, Faculty of General Studies and Foundation, University College of MAIWP International, Kuala Lumpur, Malaysia

##### **Correspondence:** Harris Shah Abd Hamid (drharris@ucmi.edu.my)

*BMC Proceedings 2024*, **18(9):**O7

**Background**: High-quality assessment is necessary for continuous improvements in teaching and learning. Rasch model has been applied widely to analyse language tests. However, its use in evaluating psychometric properties of English courses focusing on communication aspects in Malaysia is limited. The aim of this study is to evaluate the psychometric properties of the Multiple-Choice Questions part of the final examination for a college-level compulsory English course, namely Communication English.

**Materials and methods:** The answers from students taking those examinations were obtained from the University’s Examination Unit and analysed using Winstep. The exam paper covered 13 topics including listening effectively, conversation skills, tenses, and writing sentences across 30 items. All 326 answer forms were selected, and one student was excluded from the analysis due to a blank answer form.

**Results:** At the instrument level, the test could be improved further. The test’s person reliability (.71) and separation (1.57) point to room for improvement. The variance explained by the measure (28.1%) is below the desired cut-off point, however there is not enough evidence to show the existence of a secondary dimension. There were no floor nor ceiling effects and item difficulties targeted students’ abilities fairly. At the item level, the evidence is satisfactory for the quality of the items. Outfit MNSQ ranges from .69 to 1.53 with 13 items having z-standard exceeding 2.0. Item polarity is all positive, ranging from .05 to .56. Additionally, the distractors are functioning well.

**Conclusion:** While the individual items performed well, the way they are combined in a test needs to be revisited. More specifically, the dimensionality of the test points to the need to relook at the topics and re-examine how they contribute to the mastery of using English for communication purposes.

## O8 Measuring the readiness of the undergraduate internship students’ for interprofessional education at De La Salle Medical and Health Sciences Institute

### Denise Anne Q. Rellosa, Angelo R. Quiton, Angelica Gail R. Rius, Julliana Marie T. Saclayan, Amapola DG. Puaso

#### College of Medical Laboratory Science, De La Salle Medical and Health Sciences Institute, Dasmarinas Cavite, Philippines

##### **Correspondence:** Denise Anne Q. Rellosa (deniserellosabsmls32@gmail.com)

*BMC Proceedings 2024*, **18(9):**O8

**Background:** Interprofessional education (IPE) emphasizes collaborative learning between allied health students and professionals from different disciplines to strengthen teamwork and achieve the highest quality of care. In view of this, IPE is deemed a crucial factor in the interplay of healthcare professionals, patient care outcomes, and the overall healthcare delivery process. This study measures the readiness of the De La Salle Medical and Health Sciences Institute undergraduate interns for Interprofessional Education. In this cross-sectional study, a total of 165 fourth-year student interns participated from March to May 2023.

**Materials and methods:** A questionnaire titled “Readiness for Interprofessional Learning Scale” (RIPLS) was adapted and used for this study. This adapted RIPLS questionnaire was given to the research participants through online platforms and face-to-face surveys.

**Results:** The selection of the sample population was conducted using the quota sampling technique, and the findings of the study were conveyed through the following statistical measures: overall mean and standard deviation, independent t-test, and one-way analysis of variance. Results revealed that the DLSMHSI interns scored a weighted mean of 4.86 (SD=0.26) for teamwork and collaboration, 4.51 (SD=0.49) for professional identity, and 3.91 (SD=1.43) for roles and responsibilities. Additionally, it was revealed that the program significantly influences professional identity (t=2.46, *p*=0.0202) and has a profound effect on roles and responsibilities (t=42.81, *p*= <0.0001).

**Conclusion:** The findings indicate that the interns are fully prepared in terms of teamwork, collaboration and professional Identity. However, the interns are approaching readiness in terms of roles and responsibilities.

## O9 Effect of project-based learning on undergraduates’ grammar mastery

### Adiba Zailan^1^, Yong Mei Fung^2^

#### ^1^Department of Languages, Faculty of General Studies and Foundation, University College of MAIWP International, Kuala Lumpur, Malaysia; ^2^Department of English, Faculty of Modern Languages and Communication, Universiti Putra Malaysia, Serdang, Selangor, Malaysia

##### **Correspondence:** Adiba Zailan (adiba@ucmi.edu.my)

*BMC Proceedings 2024*, **18(9):**O9

**Background**: Grammar plays an important part in language learning. A lack of grammar mastery will lead to poor language performance. Many instructors often apply a traditional lecture-based approach in teaching grammar rather than giving opportunity for learners to engage with and facilitate one another to improve their grammar accuracy. Given this situation, the objective of this study is to investigate the effect of project-based learning methods on undergraduates’ grammar mastery. It also seeks to examine the undergraduates’ experiences of using project-based learning methods to improve their grammar.

**Materials and methods:** The sample of the study consisted of an intact class of 31 undergraduate students undergoing a compulsory course of Grammar for Communicative Purposes. The study adopted a quasi-experimental design which uses mixed methods data collection. Without the presence of a control group, quantitative data is acquired using non-randomized pre- and post-test writing scores. Qualitative data were gathered from semi-structured interviews and students’ reflections.

**Results:** The findings of the study showed a significant difference between the pre-test and post-test writing scores after being taught using the PBL teaching strategy (t (30) = -3.836, *p*=.004). It can be interpreted that by using the PBL teaching strategy the participants have managed to improve their grammar mastery. Meanwhile, the findings from the qualitative data showed that participants had a favourable overall experience with the project-based learning approach in the grammar classroom.

**Conclusion:** The study is expected to enlighten educators’ efforts in finding effective ways to use project-based learning in teaching grammar. It also sheds insights into learners’ experiences through project-based learning to enhance their grammar skills and communication.

## O10 Work passion and organizational citizenship behavior among academicians in Malaysia

### Amalina Ibrahim, Junaidah Yusof, Wan Mohd Azam Wan Yunus, Ana Haziqah A Rashid, Nur Syafiqah A Rahim, Yusma Fariza Yasin, Mas Idayu Saidi

#### School of Human Resource Development & Psychology, Faculty of Social Sciences & Humanities, Universiti Teknologi Malaysia, Johor Bahru, Malaysia

##### **Correspondence:** Junaidah Yusof (junaidahy@utm.my)

*BMC Proceedings 2024*, **18(9):**O10

**Background**: Nowadays, the roles and responsibilities of an academician are challenging. In Higher Education institutions, their roles are not limited to teaching and learning, but they need to be involved in research, publication, supervision, and community services. Academician must also actively participate in all programs in their organization. This condition might affect the emotion and motivation among academicians. Therefore, organizational citizenship behavior (OCB) is crucial to ensure the responsibilities are achievable throughout the year. Moreover, work passion is also important in maintaining motivation and OCB. Previous studies have mentioned that work passion is one of the factors in determining work performance, and increasing the OCB. Hence, this conceptual paper will be focused on the effect of work passion toward OCB among academicians in Malaysia. Furthermore, Self-Determination Theory, Planned Behavior Theory, and Dualistic Model of Passion also will be discussed with relevant previous studies.

**Materials and methods:** This conceptual paper has implemented the systematic literature review in work passion and OCB among academicians.

**Results:** The discussion has revealed that academicians with high work passion will lead to high OCB and support the programs in the university. In addition, through work passion, they will be internalized in their job and encourage extra-role performance such as OCB.

**Conclusion:** This conceptual paper has provided an insightful discussion on work passion and OCB among academicians, and suggested that further study must be conducted as it is important to support academicians and institutions facing the challenges in the future.

## O11 Promoting well-being in academia: a Progressive Muscle Relaxation & Breathing Exercises Training Module (PMR+BE) development

### Syazwina Muhammad Khir, Wan Mohd Azam Wan Mohd Yunus, Norashikin Mahmud, Mohammad Saipol Mohd Sukor

#### Department of Psychology, School of Human Resource Development and Psychology, Faculty of Social Sciences and Humanities, Universiti Teknologi Malaysia, Johor, Malaysia

##### **Correspondence:** Syazwina Muhammad Khir (syazwinakhir@yahoo.com)

*BMC Proceedings 2024*, **18(9):**O11

**Background**: There is an urgent need to address the rise in mental health issues such as stress and anxiety among academicians in higher educational institutions. However, the availability of evidence-based, quick, and accessible stress and anxiety prevention programs for this population is sparse. This article discusses the development and validation of the Progressive Muscle Relaxation & Breathing Exercises (PMR+BE) Training Module for university academicians.

**Materials and methods:** This module contains key components including an introduction to all related variables, General precautions, Progressive Muscle Relaxation (PMR) training procedures and scripts, and PMR training with breathing exercises (PMR+BE) procedures and scripts. Both trainings comprise specific procedures involving the following muscle groups: right & left hands, right & left arms, face, neck, shoulders, stomach, back, hips and buttocks, right and left legs, and right & left feet.

**Results:** The validation of the module was assessed by three expert panels; an associate professor in health psychology, a senior practicing clinical psychologist, and a senior academician in sports science. The validity value of the overall quality of the module content was 0.83. This article describes the content and systematic process to create and validate a practical stress and anxiety module before it is implemented in actual studies.

**Conclusion:** Future research is needed to assess the acceptability, feasibility, utility, and efficacy of the Progressive Muscle Relaxation & Breathing Exercises (PMR+BE) training module for academicians. Its potential use in higher education institutions could be a key step towards improving the academician’s mental wellbeing.

**Acknowledgement:** This research was funded by the Ministry of Higher Education (MOHE) through Fundamental Research Grant Scheme (FRGS) under the grant number FRGS/1/2020/SS0/UTM/02/23.

## O12 Resilience, academic year, and depression: a moderator analysis in a Malaysian University

### Mohamad Firdaus bin Zulkepli^1^, Amalina Ibrahim^1^, Wan Mohd Azam Wan Mohd Yunus^1,2,3^, Syazwina Muhammad Khir^1^, Mohd Nasir Masroom^1^, Nurul Farhana Mohd Noordin^1^

#### ^1^Department of Psychology, School of Human Resource Development and Psychology, Faculty of Social Sciences and Humanities, Universiti Teknologi Malaysia, Johor, Malaysia; ^2^Research Centre for Child Psychiatry, Institute of Clinical Medicine, Faculty of Medicine, University of Turku, Turku, Finland; ^3^INVEST Research Flagship Center, University of Turku, Turku, Finland

##### **Correspondence:** Wan Mohd Azam Wan Mohd Yunus (wmohdazam@utm.my)

*BMC Proceedings 2024*, **18(9):**O12

**Background**: Depression is increasingly prevalent nowadays, with students being particularly vulnerable to this mental health challenge. Concurrently, resilience is considered a pivotal protective factor, potentially shielding students from daily challenges and mitigating the factors linked to depression. Our study aimed to evaluate the influence of resilience toward depression symptoms, with academic year as the potential moderating factor among Malaysian university students.

**Materials and methods:** A cross-sectional study among 301 university students was conducted using the 20-item Centre for Epidemiological Studies Depression Scale (CES-D) and the resilience measure. The research data was analysed using SPSS for descriptive data and AMOS software was used for inferential analysis.

**Results:** Findings from this study revealed that there is a high level of resilience and a moderate level of depression symptoms. Furthermore, it showed that resilience has a significant negative influence towards depression. Additionally, it was found that the academic year does not significantly moderate the influence of resilience towards depression. However, the results show that the model has achieved a good model fit with relative chi-square = 2.36, GFI = 0.92, CFI = 0.93, TLI = 0.91, and RMSEA = 0.07.

**Conclusion:** Given the findings that the academic year did not moderate the influence of resilience towards depression, future research should explore other potential moderating variables such as social support, coping mechanisms, or extracurricular involvement to address the link between resilience and depression.

## O13 Navigating early childhood education in the digital age: lessons from the prophetic tradition

### Mohd Shawani Ahmad Sabri^1^, Ahmad Salahuddin M. Azizan^2^, Muhammad Akmalludin Mohd Hamdan^3^

#### ^1^Department of Education and Psychology, Faculty of Management, Education, and Humanities, University College of MAIWP International, Kuala Lumpur, Malaysia; ^2^Department of Languages, Faculty of General Studies and Foundation, University College of MAIWP International, Kuala Lumpur, Malaysia; ^3^Universiti Sains Islam Malaysia, Nilai, Negeri Sembilan, Malaysia

##### **Correspondence:** Mohd Shawani Ahmad Sabri (shawani@ucmi.edu.my)

*BMC Proceedings 2024*, **18(9):**O13

**Background**: In the digital age, early childhood education faces unique challenges, and the role of father figures in this context is crucial. This study explores Prophetic guidance to navigate these challenges, emphasizing their timeless relevance in shaping morally grounded children.

**Materials and methods:** In this study, a qualitative research methodology was utilised, specifically library analysis, to extract relevant Prophetic teachings and traditions pertaining to the role of father figures in early childhood education. The analysis focused on locating and interpreting relevant hadiths (Prophetic sayings and actions) from Islamic literature.

**Results:** Analysis of Prophetic traditions yielded valuable insights into the role of father figures in early childhood education within the context of the digital age. These traditions provided a theoretical and practical framework for guiding fathers in their educational responsibilities. The results underscore the significance of father figures as educational role models and their responsibilities in nurturing children’s moral and ethical development in today’s technologically driven society.

**Conclusion:** In conclusion, this study underscores the timeless relevance of Prophetic teachings in guiding father figures as they navigate the challenges of early childhood education in the digital age. The guidance provided by these traditions serves as a valuable resource for fathers seeking to fulfil their roles as educators and mentors in shaping the character and values of the next generation. By incorporating Prophetic principles into their parenting approach, fathers can play a vital role in nurturing children who are not only technologically savvy but also morally grounded.

## O14 Social media usage as a predictor of materialism: a study on Malaysian University students

### Tiffany Puah Shu Yen, Law Mei Yui

#### Tunku Abdul Rahman University of Management and Technology, Kuala Lumpur, Malaysia

##### **Correspondence:** Law Mei Yui (lawmy@tarc.edu.my)

*BMC Proceedings 2024*, **18(9):**O14

**Background**: Social media has become one of the cost-effective advertising mediums among businesses to advertise their products. These advertisements have influenced youths to make purchases with materialism being the main contributor that prompts purchasing desires. This study aims to investigate the duration of social media use per day among average university students; social media usage as a predictor of materialism; and gender differences in the duration of social media usage.

**Materials and methods:** A quantitative cross-sectional study was conducted among 100 undergraduate students (50 males and 50 females), aged 18-26 from a private university in Kuala Lumpur, through quota and snowball samplings. A Google form survey that includes Social Media Use Questionnaires (SMUQ) and Materialism Values Scale (MVS) was administered to collect data.

**Results:** The findings of this study uncovered that university students spent an average of 6 hours on social media per day (M=5.79, SD=3.06). Moreover, it was found that social media usage significantly predicts materialism F (1, 98) = 6.99, *p*<.05, = .07. Lastly, it was found that females (M=6.42, SD=2.82) spent significantly more time than males (M=5.16, SD=3.18), with t-value = -2.09, *p*<.05.

**Conclusion:** It is recommended that students should be extra vigilant about materialistic advertisements on social media to avoid falling into a materialistic mindset.

## O15 Job insecurity and turnover intention: mediating role of trust

### Rabeatul Husna Abdull Rahman^1^, Mikkay Wong Ei Leen^2^, Halimah Mohd Yusof^1^

#### ^1^School of Human Resource Development and Psychology, Faculty of Social Science and Humanities, Universiti Teknologi Malaysia Skudai, Malaysia; ^2^Department of Business Analytics, Sunway Business School, Sunway University, Malaysia

##### **Correspondence:** Rabeatul Husna Abdull Rahman (rabeatulhusna@utm.my)

*BMC Proceedings 2024*, **18(9):**O15

**Background**: Evidence from past studies has shown that perceptions of job insecurity led to different outcomes, including reduced commitment. The lack of job security can also possibly result in distrust towards the organization. Employees who have low trust in their organization and low job insecurity are alleged to have a higher intention to leave. This study investigates job insecurity and trust as the antecedents of turnover intention and utilizes trust as the mediator in the relationship between job insecurity and turnover intention.

**Materials and methods:** The data for this study was obtained through a quantitative approach, utilizing a questionnaire survey that was disseminated through online platforms. 208 employees have taken part in this cross-sectional study. The data was analysed using the PLS software.

**Results:** The majority of the respondents were male (118, 56.7%), aged between 30 and 39 years old (100, 48.1%), had bachelor’s degrees (113, 54.3%), earned above RM4,000 monthly (114, 54.9%), and worked in the private sector (113, 54.3%). The findings revealed a negative and significant relationship between job insecurity and trust, as well as between trust and turnover intention. Job insecurity is positively correlated with turnover intention, and trust mediated the linkage between job insecurity and turnover intention.

**Conclusion:** Based on these results, it can be implied that employees are likely to leave their organizations when there is a lack of job security and low trust in the organization.

## O16 Socio-demographic forces at play: examining their impact on well-being in Malaysia

### Halimah Mohd Yusof^1^, Nerisha Sarah Nur Malek^1^, Rabeatul Husna Abdull Rahman^1^, Fadillah Ismail^2^

#### ^1^Universiti Teknologi Malaysia Skudai, Johor, Malaysia; ^2^Universiti Tun Hussein Onn Malaysia, Johor, Malaysia

##### **Correspondence:** Halimah Mohd Yusof (halimahmy@utm.my)

*BMC Proceedings 2024*, **18(9):**O16

**Background**: The quality of an individual’s life is significantly impacted by their well-being, which can be subject to influence from a range of social, demographic, and economic aspects. Within the specific setting of Malaysia, there is a notable lack of research that examines the impact of socio-demographic factors psychological and social well-being of its population. This can be primarily attributed to the limited availability of measures that are specifically designed to suit the unique cultural and societal characteristics of Malaysia. The primary objective of this study was to develop a thorough understanding of psychological and social well-being among the Malaysian population.

**Materials and methods:** This study involved a sample size of 382 participants through snowball sampling focusing on Malaysians aged 20 and over, who were assessed on various measures related to psychological well-being, the social well-being index, and negative emotional symptoms.

**Results:** The regression model showed that demographic characteristics predicted psychological, negative emotional, and social well-being. Our study concluded that several demographic characteristics affect psychological, negative emotional, and social well-being. Our research found significant relationships between household income, educational achievement, and psychological well-being. Age, number of dependents, and household income were also linked to unpleasant emotional symptoms. However, gender, education, and household income were associated with social well-being.

**Conclusion:** The findings have significant consequences and provide vital insights for stakeholders responsible for developing interventions to enhance the well-being of Malaysians. The aforementioned findings underscore the importance of implementing customized interventions for persons who exhibit characteristics such as younger age, poorer educational attainment, and reduced incomes.

**Acknowledgement**

This work was supported by the Ministry of Higher Education under Fundamental Research Grant Scheme (FRGS/1/2019/SS06/UTM/02/8).

## O17 Has leader psychologically empowered nurses?

### Junaidah Yusof^1^, Amalina Ibrahim^1^, Nur Indah Sukir^2^, Ana Haziqah A Rashid^1^, Nur Syafiqah A Rahim^1^, Yusma Fariza Yasin^1^, Mas Idayu Saidi^1^, Annette d’Arqom^3^

#### ^1^School of Human Resource Development and Psychology, Faculty of Social Sciences and Humanities, Universiti Teknologi Malaysia, Malaysia; ^2^Department of Psychology, School of Medicine, International Medical University, Malaysia; ^3^Department of Anatomy, Histology, and Pharmacology, Faculty of Medicine, Universitas Airlangga, Surabaya, Indonesia

##### **Correspondence:** Amalina Ibrahim (amalina.ibrahim@utm.my)

*BMC Proceedings 2024*, **18(9):**O17

**Background**: COVID-19 globally impacted Malaysia’s society, economic, and workflow. The hospital’s main concerns were uncertainty, new working norms, and exhaustion when confronted with this situation. As most nurses in Malaysia are women, they are not just overburdened by the hospital’s workload during the pandemic, but nurses also deal with family issues. Hence, it is hoped that psychological empowerment given by leaders while working in the hospital will lessen the strain on nurses.

**Materials and methods:** This study of 461 Malaysian nurses used multi-stage cluster sampling to investigate four psychological empowerment dimensions (i.e., meaningfulness, competence, self-determination, and impact). The cluster sampling was operationalized through four stages. First, the nurse population was divided into geographical locations in Malaysia. Second, the selection of representative states for each region was used via simple random sampling. Third, the identification and selection of hospitals in each of the selected states were carried out. The fourth stage involved the selection of sample nurses in each of the selected hospitals. This study was sponsored under Matching Grant UTM and UNAIR (Reference No: PY/2021/02555).

**Results:** The findings indicate that all four dimensions of psychological empowerment are at an average level, including meaningfulness (M=4.73), competence (M=4.69), self-determination (M=4.39), and impact (M=4.27).

**Conclusion:** A leader’s empowerment behaviour is a cause of an employee’s perception of psychological empowerment. This means that a leader who promotes authority delegation and the development of employees’ abilities increases employees’ psychological empowerment at work. Therefore, the findings could help human resource professionals understand how psychological empowerment affects hospital workflow.

## O18 Building a resilient workforce: the relationship between employer branding and employee retention

### Ong Yee Hwa, Nur Syafiqah A Rahim, Mas Idayu Saidi, Ana Haziqah A Rashid, Junaidah Yusof, Amalina Saidi, Irza Hanie Abu Samah

#### School of Human Resources Development & Psychology, Faculty of Social Sciences & Humanities, Universiti Teknologi Malaysia, Johor, Malaysia

##### **Correspondence:** Nur Syafiqah A Rahim (nursyafiqaharahim@utm.my)

*BMC Proceedings 2024*, **18(9):**O18

**Background**: As businesses may face fierce competition to hire or retain talented and competent individuals to achieve organisational goals, employee retention is a crucial subject at present. With the rise of remote work, organizations have had to adapt their employer branding strategies to appeal to remote or hybrid workforces. Hence, understanding how remote work impacts employee retention and how employer branding can support this shift is an evolving area of research.

**Materials and methods:** This study has utilised quantitative methods through the distribution of questionnaires to 200 employees in one manufacturing company in Malaysia. The questionnaire used to measure factors in employer branding and employee retention was adopted and consisted of 34 items. Inferential statistics (Pearson Correlation) were employed to investigate the relationship between both variables.

**Results:** The findings show that out of five dimensions only four dimensions of employer branding have a positive relationship towards employee retention which are healthy work atmosphere (*p*=0.000), training and development (*p*=0.000), ethics and corporate social responsibility (*p*=0.000) and compensation and benefits (*p*=0.000). Only the dimension of work-life balance did not have any relationship to employee retention.

**Conclusion:** Organizations that invest in creating and nurturing a compelling employer brand are not only better positioned to attract top talent but also to retain and empower their workforce, leading to increased loyalty, satisfaction, and ultimately, a more resilient and successful organization.

## O19 Religious compliance among Muslim women entrepreneurs in the health product industry in Malaysia

### Yusma Fariza Yasin^1^, Nur Syafiqah A Rahim^1^, Nur Indah Sukir^2^, Ana Haziqah A Rashid^1^, Amalina Ibrahim^1^, Junaidah Yusof^1^, Mas Idayu Saidi^1^

#### ^1^School of Human Resource Development and Psychology, Faculty of Social Sciences and Humanities, Universiti Teknologi Malaysia, Johor, Malaysia; ^2^International Medical University, Bukit Jalil, Kuala Lumpur, Malaysia

##### **Correspondence:** Nur Syafiqah A Rahim (nursyafiqaharahim@utm.my)

*BMC Proceedings 2024*, **18(9):**O19

**Background**: Muslim women entrepreneurs who work on health products undoubtedly face a variety of difficulties and must overcome all barriers in the commercial sector. Nevertheless, the difficulty of becoming a prosperous Muslim businessperson is not only measured in terms of the amount of money made from the business but also in terms of the necessity to uphold Islamic principles.

**Materials and methods**: Interviews with ten Muslim women business owners who produce health goods in Malaysia were conducted for this paper. The interviews were semi-structured. The chosen interviewees must be Muslim women business owners.

**Results**: The findings indicate that Muslim women business owners in Malaysia who work on health products regularly incorporate their religion into their daily lives. Among the rituals observed are the following: performing five obligatory daily prayers; paying zakat; fasting throughout the month of Ramadan; performing the Hajj to Mecca once in a lifetime for those who are able; and practising asceticism in daily life by observing the “amar makruf” and “nahi munkar”.

**Conclusion**: It has been established through some activities that all interviewees also include religious observance in their daily lives. This demonstrates that despite their hectic schedules, they continue to fulfil their duties as the caliphs of Allah SWT on a daily basis.

## O20 Enhancing social support management strategies to mitigate turnover intention among employees in the retail industry

### Leong Pui Yee, Mas Idayu Saidi, Ana Haziqah A Rashid, Amalina Ibrahim, Junaidah Yusof, Nur Syafiqah A Rahim, Yusma Fariza Yasin, Mohamed Ayyub Hassan

#### School of Human Resources Development and Psychology, Faculty of Social Sciences & Humanities, Universiti Teknologi Malaysia, Johor, Malaysia

##### **Correspondence:** Ana Haziqah A Rashid (anahaziqah@utm.my)

*BMC Proceedings 2024*, **18(9):**O20

**Background**: A high turnover rate in Malaysia, especially in the food and retail industries creates several issues, such as a lack of competent staff, reduced productivity, money loss due to competition, and expensive hiring and training expenses. Since employees are one of the most valuable resources, many organizations are actively looking for ways to reduce employee turnover including considering better social support. Therefore, the aim of this paper is to determine the relationship between social support and turnover intention among retail employees.

**Materials and methods:** The data for this paper was collected using a self-administered questionnaire involving 104 employees at one retail industry located in Kuala Lumpur. The data collected were analysed using SPSS. Descriptive analysis was used to analyse the demographic profiles of respondents and the level of social support and turnover intention. ANOVA was adopted to identify the difference between years of working experience and turnover intention while Pearson Correlation was used to examine the relationship between social support and turnover intention.

**Results:** The findings show that the level of turnover intention in Company X was at a moderate level. The results indicated that there is a significant difference between years of working experience and turnover intention in Company X. Meanwhile, Pearson Correlation analysis revealed that there was a significant but negative relationship between social support and turnover intention.

**Conclusion:** These findings highlighted the perception of employees towards different dimensions of social support and how it can affect their intention to leave the organization.

## O21 Analyzing the relationship of digital literacy on manufacturing employee performance in Malaysia

### Dayang Siti Maisyarah, Ana Haziqah A Rashid, Mas Idayu Saidi, Amalina Ibrahim, Nur Syafiqah A Rahim, Junaidah Yusof, Yusma Fariza Yasin

#### School of Human Resources Development & Psychology, Faculty of Social Sciences & Humanities, Universiti Teknologi Malaysia, Johor, Malaysia

##### **Correspondence:** Mas Idayu Saidi (masidayu@utm.my)

*BMC Proceedings 2024*, **18(9):**O21

**Background**: Industrial Revolution 4.0 has indicated the beginning of changes in all industries due to technological improvements. The use of technology in the workplace is expected to increase at a faster speed with the advent of digital technology expecting employees with high digital literacy. Yet, many employees have difficulty coping with and keeping up with technical expertise to complete their work. The lack of digital literacy might affect the employees’ performance. Hence, it is significant to investigate the relationship between digital literacy and employee performance among manufacturing employees in Malaysia.

**Methods:** This study has utilised a quantitative research design with a convenient sampling approach. A total of 85 questionnaires were distributed among employees in administrative positions within a manufacturing company based in Malaysia. The questionnaire used to measure employee digital literacy and performance was adopted and consisted of 46 items. Descriptive (Mean) and inferential statistics (Spearman Correlation) were employed to investigate the relationship between both variables.

**Results:** The findings highlight a high level of digital literacy (M=3.97) and a high level of employee performance (M=4.01) among Malaysian manufacturing company’s employees. The findings showed a positive and strong relationship between digital literacy and employee performance among manufacturing employees *r*=0.749 (*p*<0.001).

**Conclusion:** Overall, this research underscores the significance of an employee’s digital literacy skills in shaping their performance, particularly within the manufacturing industry in Malaysia. Thus, the findings could assist the employers in manufacturing industry in enhancing the digital literacy skills of their employees to maximize employee performance.

## O22 Establishing a comprehensive “Halal Online Food Delivery” (HOFD) in Malaysia

### Khazizul Maulod Pahim^1^, Mohd Lutfi Iskandar Sahid^2^, Zunirah Talib^3^

#### ^1^Department of Management, Faculty of Management, Education, and Humanities, University College of MAIWP International, Kuala Lumpur, Malaysia; ^2^Kuala Lumpur Metropolitan University College, Kuala Lumpur, Malaysia; ^3^Management Science University, Kuala Lumpur, Malaysia

##### **Correspondence:** Khazizul Maulod Pahim (khazizulmaulod@ucmi.edu.my)

*BMC Proceedings 2024*, **18(9):**O22

**Background**: There is an increasing demand for the food and beverages industry in Malaysia especially during the COVID-19 Pandemic due to restrictions on dining in or eating out in eateries such as restaurants, café, and stalls. Thus, online food ordering has become the new normal. Not only in Malaysia, but Online Food Delivery (OFD) has become a trend and is very popular among the younger generation in the whole world. Despite the importance and the changing consumer behaviour towards OFD services in Malaysia, the existence of other Online Delivery Platform (ODP) also has tremendous demand as people are likely to shop using online platforms such as Foodpanda, GrabFood, Lalamove, SmartBite, AirAsia Food etc.

**Materials and methods:** This study utilised a quantitative method. The questionnaires were distributed to all OFD stakeholders comprising all riders, restaurants, customers, and government bodies involved in the OFD industries within Malaysia especially those who handle Halal foods.

**Results:** From this study, the new enhanced framework for “Halal Online Food Delivery (HOFD)” has been introduced.

**Conclusion:** The findings of this paper will help the HOFD operator to clearly understand and practice the Standard Operation Procedure (SOP) for delivering Halal food.

## O23 Chromatographic analysis of Ganciclovir assisted by green solvent technique

### Archana Mandava, Sumithra Mani

#### School of Pharmaceutical Sciences, Vels Institute of Science, Technology and Advanced Studies, Chennai Tamil Nadu, India

##### **Correspondence:** Archana Mandava (archana.drf@gmail.com)

*BMC Proceedings 2024*, **18(9):**O23

**Background**: Green analytical techniques can provide several benefits to environmental analysis, including enhancing the quality and reliability of findings, improving the safety and health of analysts, reducing expenses and resource consumption, and promoting social and environmental responsibility. Ganciclovir was quantified through chromatographic analysis using a mobile phase consisting of acetonitrile, ion-pairing agents, buffers, etc., which are not environmentally friendly.

**Materials and methods:** The initial estimation of Ganciclovir was conducted using phosphoric acid at pH 3.0, and the mobile phase was optimized, consisting of ethanol (a green solvent) and acidic water mixed in a ratio of 80:20% v/v. The solutions were chromatographed at a constant flow rate of 1.0 ml/min.

**Results:** The linearity range was determined to be 10-50 μg/ml. The linear regression coefficient was not greater than 0.999. The % RSD values were < 2%, indicating the accuracy and precision of the method. The percentage recovery ranged from 98% to 102% for ganciclovir. LOD and LOQ were within the specified limits.

**Conclusion:** The developed green solvent-assisted method offers a reliable and environmentally friendly approach for the analysis of ganciclovir in bulk and pharmaceutical dosage forms. This method exhibits good selectivity, linearity, accuracy, precision, robustness, and system suitability. It can be employed in the routine analysis and quality control of pharmaceutical products containing ganciclovir, ensuring their safety and efficacy.

## O24 Imeglimin: unveiling the potential of a cutting-edge antidiabetic medication - a review

### Syed Shah, Archana Mandava

#### Department of Pharmaceutical Chemistry and Analysis, School of Pharmaceutical Sciences, Vels Institute of Science, Technology and Advanced Sciences, Chennai, Tamil Nadu, India

##### **Correspondence:** Archana Mandava (archana.sps@velsuniv.ac.in)

*BMC Proceedings 2024*, **18(9):**O24

**Background**: Imeglimin, is the oral tetrahydrotriazine-containing compound, which is the first molecule from the new class of drugs – the glimins. It is an antidiabetic medication which is administered orally, and used in type 2 diabetes mellitus treatment and diagnosis. In Japan in 2021, Imeglimin was first approved for its clinical use as an oral antidiabetic medication. Poxel SA focused on the development of imeglimin as an antidiabetic medication and conducted clinical trials which gave positive results in patients with diabetes.

**Materials and methods**: Imeglimin has dual effects, they are; to reverse pancreatic β-cell dysfunction and to increase insulin activity. Imeglimin’s unique mode of action, which targets two critical diabetes-related weaknesses, namely insulin resistance and beta-cell function, as well as its safety profile, may be able to assist patients in better control of their condition.

**Results**: The outcomes of the clinical studies support the strong effectiveness and positive safety findings from the Phase 2b study in Japan and the potential advantages that Imeglimin may have for type 2 diabetic patients throughout the world. Currently, Imeglimin is approved for manufacturing and marketing in Japan under the brand Twymeeg as an oral antidiabetic medication.

**Conclusion:** This review highlights the discovery, physicochemical and pharmacokinetic properties, mechanism, synthesis, marketed formulation and patents for the novel drug Imeglimin. In short, we discuss the potentials and properties of the Imeglimin a novel antidiabetic medication.

## O25 Taste masking of anti-psychotic drug using ion exchange resin

### Sriram Karuna Moorthy, Shanmugasundaram Palani

#### Department of Pharmaceutical Chemistry and Analysis, School of Pharmaceutical Sciences, Vels Institute of Science, Technology and Advance studies, Chennai, Tamil Nadu, India

##### **Correspondence:** Shanmugasundaram Palani (dean.sps@velsuniv.ac.in)

*BMC Proceedings 2024*, **18(9):**O25

**Background**: The primary goal of this review paper is to discuss various aspects of using ion exchange resins to disguise the taste of bitter medications. Ion exchange resin complexes, which may be made from both acidic and basic medicines, have received a lot of attention and are now commercially available.

**Materials and methods**: Salts of cationic and anionic exchange resins are insoluble complexes in which bound drug ions are exchanged with ions ordinarily present in bodily fluids, resulting in drug release. Ion exchange resins are employed for a variety of drug delivery and therapeutic applications due to their versatility. Polymers with properly substituted acidic groups, such as carboxylic and sulfonic for cation exchangers, or basic groups, such as quaternary ammonium for anion exchangers, are employed as resins. For taste masking, synthetic ion exchange resins have been utilized in pharmacy and medicine.

**Results**: The bitterness of pharmaceutical medicines is important for patient compliance since bitter drugs’ oral administration is sometimes hampered which leads to non-compliance. IER is one of the most often used methods for disguising the taste of bitter medications. Depending on the nature of the medicine, weak cation exchange or weak anion exchange resins are employed to conceal the taste. The drug resin compound is completely tasteless and has no aftertaste, yet its bioavailability is unaffected.

**Conclusion**: IER has been found to be equally suited for drug delivery strategies such as controlled release, transdermal, nasal, topical, and taste masking in recent research.

## O26 Quality by design perspective for development and validation of RP-HPLC method for simultaneous estimation of Benidipine and Telmisartan in Bulk and tablet dosage form

### Pavithra Devi Mani, Sudha Thomas

#### Department of Pharmaceutical Chemistry and Analysis, School of Pharmaceutical Sciences, Vels Institute of Science, Technology and Advanced Sciences, Chennai, Tamil Nadu, India

##### **Correspondence:** Sudha Thomas (tsudha.sps@velsuniv.ac.in)

*BMC Proceedings 2024*, **18(9):**O26

**Background:** Quality by Design (QbD) focuses on consistent quality with predetermined parameters, emphasizing risk management for robust methods. Understanding factors and their interactions through systematic experiments is vital in QbD. The study outlines a risk-based HPLC method for Benidipine and Telmisartan determination in bulk and pharmaceutical forms.

**Materials and methods:** Three key components of the RP-HPLC method, organic composition of mobile phase, buffer pH, and flow rate, were optimized using a central composite design. Design Expert 12.0 software was employed to optimize chromatographic conditions. This included using a Phenomenex ODS C18 column (250 mm × 4.6 mm, 5.0 μm) with a mobile phase consisting of acetonitrile and phosphate buffer (0.9% ortho-phosphoric acid, pH 4.0) in a 65:35%v/v ratio, with a flow rate of 0.8 ml/min.

**Results:** The retention times were 7.62 min for Benidipine and 10.09 min for Telmisartan. The developed method exhibited strong linearity (r² = 0.9994 for Benidipine, 0.9992 for Telmisartan) in the respective concentration ranges. System suitability tests met the requirements for tailing factor and theoretical plates. Precision had low % RSD values (0.366 for Benidipine, 0.069 for Telmisartan). The assay showed high accuracy (100.00 ± 1.30% for Benidipine, 100.13 ± 0.35% for Telmisartan). Method validation parameters adhered to ICH guidelines.

**Conclusion:** Utilizing Design Expert 12.0 software, Central Composite Design assessed the interactions of mobile phase organic composition, buffer pH, and flow rate at three levels, examining responses like capacity factor (k1), Resolution (Rs1,2), and retention time. This enhanced understanding of chromatographic separation, bolstering confidence in the HPLC method’s efficacy. QbD methodology aided in constructing the analytical method and improving variable comprehension across levels.

## O27 A systematic review of mercaptopurine

### Nithish Kumar Gandhi, Shanmugasundaram Palani

#### Department of Pharmaceutical Chemistry and Analysis, School of Pharmaceutical Sciences, Vels Institute of Science, Technology and Advance studies, Chennai, Tamil Nadu, India

##### **Correspondence:** Shanmugasundaram Palani (dean.sps@velsuniv.ac.in)

*BMC Proceedings 2024*, **18(9):**O27

**Background:** Mercaptopurine is a medication in the class of purine antagonists. The medication is used in the management and treatment of acute lymphoblastic leukemia. Mercaptopurine is a value agent in managing and treating leukemias and inflammatory autoimmune disorders. An antimetabolite antineoplastic agent with immunosuppressant properties. It interferes with nucleic acid synthesis by inhibiting purine metabolism and is used, usually in combination with other drugs, in the treatment of or in remission maintenance programs for leukemia.

**Materials and methods:** The objective of this study is to identify the mechanism of action and administration of mercaptopurine, describe the potential adverse effects of mercaptopurine, review the appropriate monitoring for patients on mercaptopurine, summarize the interprofessional team strategies for improving care coordination and communication to advance mercaptopurine and improve outcomes.

**Results:** This article will highlight the discovery & further history, physical & chemical properties, pharmacokinetic characteristics, mechanism of action, method of synthesis, medicinal uses, adverse effects, treatment of overdose, contraindications, interaction, conventional marketed formulation, novel marketed formulation, patents other key factors (e.g., off-label uses, dosing, pharmacodynamics, pharmacokinetics, monitoring, relevant interactions).

**Conclusion:** This study is pertinent for healthcare professionals and students to indicate the usage of mercaptopurine for the welfare of the patients.

## O28 Role of flavonoids in the improvement and protection of ulcerative colitis and gastro-toxicity

### Monisha Jayaprakash^1^, Jayashree Venugopal^2^

#### ^1^Department of Pharmaceutics, School of Pharmaceutical Science, Vels Institute of Science and Technology and Advanced Studies, Chennai, Tamil Nadu, India; ^2^Department of Pharmacology, School of Pharmaceutical Science, Vels Institute of Science and Technology and Advanced Studies, Chennai, Tamil Nadu, India

##### **Correspondence:** Monisha Jayaprakash (monisha.sps@velsuniv.ac.in)

*BMC Proceedings 2024*, **18(9):**O28

**Background:** Ulcerative Colitis is characterized by mucosal inflammation initiating in the rectum. Since ancient times herbal natural therapies have been practiced worldwide for the treatment of ulcerative colitis. Medical therapy for ulcerative colitis (UC) was limited to corticosteroids. Treatment of UC remains challenging due to the limited effectiveness and severe side-effects of the currently available drugs. Flavonoids due to their anti-inflammatory and antioxidant properties exhibit gastroprotective effects against UC. The present study involves research on flavonoids which act as muco-protective and have been reported to show gastro-protective activity in UC therapy.

**Materials and methods:** The main constituent used is flavonoids along with gelling-agents, mannitol, sucrose, citric acid, and honey. The chewable gummy tablets were prepared through the Heating and Congealing method and then their physical characters were analysed.

**Results:** Prepared formulations of flavonoids using gelling agents have unique shapes, tastes and pleasing appearance. A higher gelling agent concentration has higher mechanical strength and, as a result, less elastic texture.

**Conclusion:** CGTs developed using gelling-agents are considered optimal formulations and provide a better texture which acts as a gastroprotective agent and aids in gastro-toxicity.

## O29 RP-HPLC method development and validation for simultaneous estimation of metformin and Evogliptin by DoE approach

### Ajith Uthrapathi, Sudha Thomas

#### Department of Pharmaceutical Chemistry and Analysis, Vels Institute of Science, Technology and Advance studies, Chennai, Tamil Nadu, India

##### **Correspondence:** Sudha Thomas (tsudha.sps@velsuniv.ac.in)

*BMC Proceedings 2024*, **18(9):**O29

**Background:** Quality by Design technique, experimental design (DoE) is the process of organizing experiments to ensure that the necessary information is gathered as effectively and precisely as feasible. A risk assessment study was performed, followed by a factor screening study using a fractional factorial design (FFD). Subsequently, factor optimization was conducted using a central composite design (CCD), with organic phase composition in mobile phase, Buffer pH and flow rate ratio as the critical method parameters (CMPs). Tailing factor (TF), Resolution (Rs_1,2_) and retention time (Rt_1)_ were selected as the critical analytical attributes (CAAs). In order to get the optimal solution, Polynomial modelling was performed, then followed by numerical and graphical optimization.

**Methods:** Chromatographic separation was conducted using Phenomenex ODS column C_18_ (250 mm 4.6 mm, 5.0) as stationary phase, the mobile phase composition of Acetonitrile (ACN) and ammonium acetate buffer (10 mM, pH 10.2) in the ratio of 65:35% v/v, flow rate of 1.2mL/min, column oven temperature of 40 °C and UV detection at 227 nm.

**Results:** Retention times were 2.37 min and 9.01 min for Metformin and Evogliptin, respectively. The method was found to be linear in the range of 50-250 μg/mL (R^2^ >0.9995) and 0.5-2.5μg/mL (R^2^ >0.9994) for Metformin and Evogliptin respectively. The system suitability test parameters, tailing factor and theoretical plates, were found to be within the limit. The assay was found to be 100.12 ± 0.22% and 100.23±1.23 respectively. The method validation parameters were within the prescribed limit as per ICH guidelines.

**Conclusion:** Central Composite Design optimized chromatographic conditions by assessing interactions and quadratic effects of key factors on three chosen responses. Models for screening and optimization were highly significant, affirming method predictability. This simple, accurate, and robust method effectively analyzes Metformin and Evogliptin in both bulk and pharmaceutical forms with high selectivity.

## O30 A brief review of the development and validation of analytical methods

### Ajeeth Kumar Thiyagarajan, Shanmugasundaram Palani

#### Department of Pharmaceutical Chemistry and Analysis, School of Pharmaceutical Sciences, Vels Institute of Science, Technology and Advance studies, Chennai, Tamil Nadu, India

##### **Correspondence:** Shanmugasundaram Palani (dean.sps@velsuniv.ac.in)

*BMC Proceedings 2024*, **18(9):**O30

**Background**: The creation of analytical techniques helps to comprehend the critical process factors and lessens their influence on precision and accuracy. Designing analytical methods that adhere to GMP and GLP requirements requires using the processes and acceptance criteria defined in ICH standards Q2 (R1). The steps involved in method development include defining the analytical method objectives, conducting a literature review, developing a method plan, optimizing the method, validating the method, transferring the method, and sample analysis. The method development involves the process of Wet Analysis, Chromatographic Analysis and Spectroscopical Analysis. The process of developing an analytical method entails setting up a variety of experimental circumstances for the analysis of chemical samples. The chemical components in drug products designed for commercial manufacturing can be identified, separated, quantified, and studied using developed analytical procedures.

**Materials and methods:** Feasibility, Development, and Validation were applied in this method to narrate the review.

**Results:** The development of analytical approaches facilitates understanding of the crucial process variables and reduces their impact on precision and accuracy. The procedures and acceptance standards outlined in ICH standards Q2 (R1) must be used when creating analytical methods that correspond to GMP and GLP regulations.

**Conclusion:** The development of analytical methods aids in understanding the crucial process variables and reduces their impact on precision and accuracy. The procedures and acceptance criteria outlined in the ICH standards Q2 (R1) must be utilised to design analytical methods, which should be employed following GMP and GLP requirements. The influence that the procedures have on out-of-spec rates and process capability needs to be measured and analysed once they have been designed, qualified, and validated to determine their efficacy for future use.

## O31 Effect of Apium graveolens stalk extract on the phagocytosis of Candida albicans by Polymorphonuclear Cells (PMNs) in vitro

### Job Matthew Abasola, Mary Angeline Jacildone, Leann Faith Bugaling, Emily Cres Esteban, Maria Daniella Amon

#### Department of Medical and Technology, University of Baguio, Baguio, Benguet, Philippines

##### **Correspondence:** Job Matthew Abasola (20181698@s.ubaguio.edu)

*BMC Proceedings 2024*, **18(9):**O31

**Background**: Flavonoids are known to increase the phagocytic activity of polymorphonuclear cells (PMNs). This study utilised celery, *Apium graveolens*, which is known to be highly concentrated with flavonoids. The phagocytic activity of PMNs combined with different concentrations of the stalk extract of celery was measured through their phagocytic indexes.

**Materials and methods:** The study followed a quantitative experimental design. The study utilised a micro method which estimates the killing and phagocytosis of *Candida albicans* by human PMNs developed by Wood & White. This method involved mixing equal amounts (10μL) of the different concentrations (100%, 70%, 50%) of the stalk extract, buffy coat, and *C. albicans* into tubes. A smear was then made and stained with Wright’s stain. 200 cells were then counted. The phagocytic index formula was used to compute the phagocytic activity. Kruskal Wallis test was used to analyze the data. Ethical clearance was obtained and approved. Formula: Phagocytic index = (PMNs with intracellular *C. albicans*/extracellular *C. albicans*) x (PMNs with intracellular *C. albicans*/ total PMNs counted) x 100.

**Results:** The findings showed that the effect of 100% pure crude showed the highest mean phagocytic index of 30.87, which is higher than the control (no extract) having a 6.39 phagocytic index, preceded by the 50% crude extract (phagocytic index= 24.08) and the 70% ethanol solvent (phagocytic index= 28.21). The results showed an increasing effect on the phagocytosis of *C. albicans* by PMNs in vitro upon adding *Apium graveolens* extract, thereby enhancing phagocytic activity. The presence of flavonoids in celery has an increasing effect on phagocytic activity. The PMNs in the 70% ethanol solvent group had higher *C. albicans*, evidenced by the fact that the PMNs seemed more deeply stained and included more stained *C. albicans* cells.

**Conclusion:** Comparing the effects of the various concentrations of *Apium graveolens* stalk extract on the phagocytosis of *C. albicans*, the results indicated no significant differences (*p*-value =0.276). The different concentrations of the extract enhanced the phagocytic activity of the PMNs. Still, there is no significant difference between the different concentrations of the extract regarding their effect on phagocytic activity.

## O32 *In-vitro* anti diabetic potency of ethanolic extract of leaves of *Vitex negundo* L.

### Karthick Raja Devendran, Chandru Ganesh, Gandhimathi Retnasamy

#### School of Pharmaceutical Sciences, Vels Institute of Science, Technology and Advanced Sciences, Chennai, Tamil Nadu, India

##### **Correspondence:** Karthick Raja Devendran (drgmathipharm2017@gmail.com)

*BMC Proceedings 2024*, **18(9):**O32

**Background**: Diabetes mellitus refers to a group of diseases that affect how the body uses blood sugar (glucose). Glucose is an important source of energy for the cells that make up the muscles and tissues. It is also the brain’s main source of fuel. The current investigation aims to explore the antioxidant and antidiabetic activity in the leaves of *Vitex negundo* Linn. The objective of the study is to extract the phytoconstituents from the leaves of *Vitex negundo* by Soxhlet extraction with ethanol and to perform the Anti-Oxidant Estimation by DPPH method. Besides, the study performs the assay of α- Amylase Inhibition, α- glucosidase Inhibition in-vitro studies and glucose uptake studies.

**Materials and methods**: The *Vitex negundo* leaves (1kg) were dry and so pulverized. The powder was prepared by grinding. The methods used for extraction by Soxhlet apparatus.

**Results**: The powdered sample was successfully extracted with ethanol using Soxhlet extractor. About 1.02% yield was obtained in the given leaves of *Vitex negundo* Linn. The highest tested concentration was 0.5mg of crude extract and it showed 92.4% inhibition against DPPH free radical.

**Conclusion**: The results of the current study show that *Vitex negundo* has moderate anti-diabetic activity and good antioxidant activity. The data show that *Vitex negundo* can be used as an anti-diabetic agent.

## O33 Validated spectrophotometric method for determination of Saxagliptin in Bulk and pharmaceutical dosage forms using ion pair complexation method

### Selvakanimozhi Murugan, Archana Mandava

#### Department Pharmaceutical Chemistry and Analysis, School of Pharmaceutical Sciences, Vels Institute of Science, Technology and Advance studies, Chennai, Tamil Nadu, India

##### **Correspondence:** Archana Mandava (archana.sps@velsuniv.ac.in)

*BMC Proceedings 2024*, **18(9):**O33

**Background**: Saxagliptin is a selective and potent dipeptidyl peptidase (DPP)-4 inhibitor approved as an adjunct to diet and exercise to improve glycemic control in type 2 diabetes mellitus (T2DM). In patients with T2DM, once-daily administration of saxagliptin before breakfast results in sustained inhibition of plasma DPP-4 activity and reduction of postprandial hyperglycemia, including after dinner, which is associated with an increase in plasma glucagon-like peptide-1 levels.

**Materials and methods:** In method-A, a 10ml volumetric flask, aliquots (0.2-1.0ml) of Saxagliptin standard solution were added with 0.8ml of 0.1% hydrochloric acid and 1ml of bromothymol blue was added to each flask. The volume was adjusted to 5ml with water and then extracted with 5ml chloroform and the absorbance of each solution was measured at 425nm. In method-B, a 10ml volumetric flask, aliquots (0.2-1.0ml) of Saxagliptin standard solution were added along with 2ml of Bromocresol green, and 2ml of buffer solution was added to each flask. The mixture was extracted with 10ml of chloroform. The organic phase was extracted and dehydrated with anhydrous sodium sulfate and the volume was made up to mark with chloroform. The absorbance of each solution was measured at 415nm against a reagent blank.

**Results:** The linearity range was found to be 5-23μg/ml for methods A and B. The linear regression coefficient was not more than 0.999. The % RSD values were < 2% for both methods. The percentage recovery varies from 98-102% for saxagliptin. The limit of quantification and detection was found to be within the acceptable limits.

**Conclusion:** The proposed methods do not require expensive and complex equipment. The methods are simple, fast and robust, with high accuracy and precision. BTB and BCG are inexpensive reagents and are available in any analytical laboratory. Therefore, these methods are valuable for their routine use in quality control laboratories for the analysis of saxagliptin.

## O34 Genetic diversity of Carbapenem-Resistant Pseudomonas aeruginosa (CRPA)

### Sherill Tesalona^1,2^, Maria Ruth Pineda-Cortel,^1,2,3^, Miguel Francisco Abulencia^4^, Sylvia Sapula^5^, Henrietta Venter^5^ and Evelina Lagamayo^6,7,8^

#### ^1^The Graduate School, University of Santo Tomas, Manila, Philippines; ^2^Department of Medical Technology, Faculty of Pharmacy, University of Santo Tomas, Manila, Philippines; ^3^Research Center for the Natural and Applied Sciences, University of Santo Tomas, Manila, Philippines; ^4^Bioinformatics and Data Management Unit, Research Institute for Tropical Medicine, Philippines; ^5^School of Pharmacy and Medical Sciences, University of South Australia, Adelaide, Australia; ^6^Institute of Pathology, St. Luke’s Medical Center, Quezon City, Philippines; ^7^Institute of Pathology, Chinese General Hospital and Medical Center, Manila, Philippines; ^8^Department of Clinical Pathology, University of Santo Tomas Hospital, Manila, Philippines

##### **Correspondence:** Sherill Tesalona (sdtesalona@ust.edu.ph)

*BMC Proceedings 2024*, **18(9):**O34

**Background**: The increasing cases of Carbapenem-resistant Pseudomonas aeruginosa (CRPA) have raised significant public health concerns worldwide. The emergence of new strains made management and treatment more difficult.

**Materials and methods:** Whole-genome sequencing (WGS) technology was employed to map genes associated with antimicrobial resistance (AMR) and to identify multilocus sequence types (MLST). Twenty-nine strains of P. aeruginosa isolated from patients in three private hospitals in Manila, Philippines, were sequenced, and their AMR genes and MLST were mapped to 401 published genomes of strains originating from Asian countries with similar studies.

**Results:** The WGS analysis of strains of P. aeruginosa revealed the distinct distribution of AMR genes belonging to OXA and PDC, having 17 and 34 variants, respectively. The study revealed higher rates of the following variants: OXA186, OXA50, OXA488, OXA846, OXA494 and PDC8, PDC1, PDC35, PDC11, PDC3. Furthermore, eight biocidal-resistant genes were identified, namely Mex, Tri, Opm, Opr, PmpM, YajC, Par and sox. Mex variants were the most frequent (*n*=12) in comparison to Tri variants (*n*=3) and Opm variants (*n*=3). The MLST identified 21 novel STs and seven known STs: ST3753 (*n*=2) from hospital A, five STs from hospital B (ST357, ST1822, ST312, ST111 and ST389), and ST 175 (*n*=2) from hospital C. By integrating the MLST studies, 177 novel STs and 37 known STs were identified. Among 177 novel STs, 96 are multi-allelic at locus trpE, with alleles 7 and 321, respectively.

**Conclusion:** There is no evidence of the prevalence of high-risk clones; however, the high-level genetic diversity will facilitate the acquisition and spread of strains carrying AMR and biocidal-resistant genes, a major public health risk.

**Acknowledgment:** Sylvia Sapula, PhD and Henrietta Venter, PhD of School of Pharmacy and Medical Sciences, University of South Australia, Adelaide, Australia, for facilitating and helping us in the execution of the whole genome sequencing.

## O35 Novel substituted triazole derivatives as optimistic anti Alzheimer agent: synthesis, molecular docking, and *in-vitro* cytotoxic evaluation

### Joel Mart, Ronald Darwin

#### Department of Pharmacology, School of Pharmaceutical Sciences, Vels Institute of Science, Technology and Advanced Sciences, Chennai, Tamil Nadu, India

##### **Correspondence:** Joel Mart (joelmart.sps@velsuniv.ac.in)

*BMC Proceedings 2024*, **18(9):**O35

**Background:** Alzheimer’s disease (AD) is becoming a more serious public health issue as a result of our population’s rising life expectancy. Finding a means of stopping and delaying the sickness is urgently needed. The present study aims to determine if new synthetic analog Triazole derivatives may protect against human SH-SY5Y neuroblastoma cells.

**Materials and methods:** The designed triazole derivatives were synthesized and characterized using different analytical techniques such as FT-IR, 13C NMR, 1H NMR, and Mass spectroscopy. In silico molecular docking studies were performed using lip dock and Discovery studio software to find the interaction between protein and ligand and calculate the binding energy. The in vitro cytotoxicity of the synthesized compounds was assessed against the human SH-SY5Y neuroblastoma cell line using an MTT assay.

**Results:** In molecular docking studies the synthesized triazole derivative compound TM and DOX showed good binding energy -16.33 and -16.92. The IC 50 value of the synthesized compound DOX in the human SH-SY5Y neuroblastoma cell line was 23.14 μg/ml.

**Conclusion:** The in vitro studies confirmed that triazole derivative compounds had lower cytotoxicity values. Molecular docking studies compound showed a good agreement with the obtained pharmaco-biological results.

## O36 Overview on antiepiletic drugs and how cenobamate differ from other antiepiletic drugs

### Kajhareshevarmaa Rameshkaanth, Gandhimathi Retnasamy

#### Department of Pharmaceutical Chemistry and Analysis, School of Pharmaceutical Sciences, Vels Institute of Science, Technology and Advanced Sciences, Chennai, Tamil Nadu, India

##### **Correspondence:** Gandhimathi Retnasamy (drgmathipharm2017@gmail.com)

*BMC Proceedings 2024*, **18(9):**O36

**Background**: Since 1955, numerous alkyl-carbamates, including meprobamate, flupirtine, felbamate, retigabine, carisbamate, and cenobamate, have been created for the treatment of anxiety and epilepsy. While their effectiveness as antiseizure medications has varied, they have all been plagued by the appearance of serious and occasionally fatal side effects.

**Materials and methods:** We analyze and contrast their main molecular mechanisms of action, antiseizure profile, and, where viable clinical efficacy in this review. For comparison, the archetypal -aminobutyric acidergic (GABAergic) modulator phenobarbital’s preclinical, clinical, and mechanistic profile is provided. All of the therapeutically approved alkyl-carbamates, like phenobarbital, are capable of increasing inhibitory neurotransmission by modulating the GABAA receptor, although the particular mechanism of interaction varies depending on the medicine. Additionally, it has been demonstrated that a number of alkyl-carbamates interact with voltage-gated ion channels.

**Results:** Flupirtine and retigabine both activate K+ currents mediated by KCNQ (Kv7) K+ channels, but felbamate, carisbamate, and cenobamate have been demonstrated to inhibit Na+ channels. Cenobamate appears to be unusual among alkyl-carbamates in its capacity to preferentially reduce the persistent rather than transient Na+ current.

**Conclusion:** Cenobamate has a unique mechanistic profile among alkyl-carbamates a unique synergy between its actions at the GABAA receptor and on persistent Na+ currents. However, the strong efficacy of cenobamate is tempered by the risk of significant rash and low tolerability at higher doses, implying that additional safety studies and clinical experience are required to assess the true clinical utility of cenobamate.

## O37 A novice formulation of marine drug loaded polymeric nanoparticle

### Velmurugan Thiyagarajan^1^, Shanmugasundaram Palani^2^

#### ^1^Department of Pharmaceutics, School of Pharmaceutical Sciences, Vels Institute of Science, Technology and Advanced Studies, Chennai, Tamil Nadu, India; ^2^Department of Pharmaceutical Chemistry and Analysis, School of Pharmaceutical Sciences, Vels Institute of Science, Technology and Advanced Studies, Chennai, Tamil Nadu, India

##### **Correspondence:** Velmurugan Thiyagarajan (samsimahe1978@gmail.com)

*BMC Proceedings 2024*, **18(9):**O37

**Background**: The present study aims to formulate and characterize green marine drug-loaded polymeric nanoparticles by nanoprecipitation technique. It improves solubility and bioavailability, reduces toxicity, enhances release and provides better formulation.

**Materials and method:** Nanoparticles containing green marine drug were prepared by Nanoprecipitation where the drug and Chitosan are dissolved in 20 mL of acetone. This organic phase is quickly injected into the aqueous phase containing 40 mL of either 1% or 2% w/v of Pluronic F68/PVA solution with moderate magnetic stirring at room temperature. The organic phase to aqueous phase ratio was 1:2. The polymeric Nanoparticles are spontaneously formed and turned the solution slightly turbid. Then, acetone is removed by continuous stirring for 3-4 hrs.

**Results:** The polymers were found to be compatible with the chosen marine drug. The particle size of nanoparticles was 190nm, and the PDI ranged from 0 to 1. The Zeta potential of the formulations showed negative zeta potential (-25mV to -29mV). The drug content was 86.75%, and the entrapment efficiencies at 76.48%.

**Conclusion:** The green marine drug-loaded nanoparticle was prepared through the nanoprecipitation method, and the formulation of 1:1 drug-to-polymer ratio showed satisfactory results in the mean particle size, polydispersity index, zeta potential and loading efficiency, and entrapment efficiency. FTIR study concluded that there was no interaction between the drug and polymers.

## O38 Extraction of polyphenols from *Vitis vinifera* and comparative study of antioxidant, antifungal and antimicrobial activity

### Hema Pachamuthu, Gandhimathi Retnasamy

#### Department of Pharmaceutical Chemistry and Analysis, School of Pharmaceutical Sciences, Vels Institute of Science, Technology and Advanced Studies, Chennai, Tamil Nadu, India

##### **Correspondence:** Gandhimathi Retnasamy (drgmathipharm2017@gmail.com)

*BMC Proceedings 2024*, **18(9):**O38

**Background**: The present study aims to extract the polyphenols from *Vitis vinifera* and determine the antioxidant activity, antifungal and antimicrobial activity of the natural polyphenols.

**Materials and methods:** The grape seed extract was prepared from *Vitis vinifera* seeds. The phytochemical constituents and the total phenolic hydrogen compound and also flavonoids were assessed using reagent *Folin Ciocalteu*, Shinoda test and NaOH method respectively. The antioxidant property of the extract was evaluated by using the nitric oxide scavenging method. The antifungal activity of the extract was determined by using a well diffusion method. The diluted fungal solutions of 15μl, 20μl, and 25μl were poured over the media to spread uniformly on the surface. The surface was little dried wells of 8mm were punched in the agar with stainless steel borer and filled with 5μl,10μl and 15μl plant extracts. The control wells containing solvents (negative control) were also run parallel in the same plate. The plates were incubated at 28°C for 72 hours and the antifungal activity was assessed by measuring the diameter of the zone of inhibition at the interval of 24 hours. The antimicrobial activity of the extracts was tested against the Gram-positive and Gram-negative bacterial strains by observing the zone of inhibition.

**Results:** The quantification was determined by the colorimetric method. Our extract contains phenolic hydroxyl flavonoids so we estimated by using FC reagent. The 𝗁 max of our colorimetry studies was 656nm. We quantified the Quercetin by colorimetric curve and the amount present in 1gm of skin and seed was estimated by solvent extraction method.

**Conclusion:** From the comparative studies the extract from *Vitis vinifera* inhibits the growth of *Aspergillus niger* during antifungal activity and also shows the antioxidant property with Griess reagent. However, it shows minimal inhibition against the Gram-positive and Gram-negative bacteria.

## O39 An overview on Niraparib – an anti-cancer drug

### Poojah Aravamudhan, Afroz Patan

#### Department of Pharmaceutical Chemistry and Analysis, School of Pharmaceutical Sciences, Vels Institute of Science, Technology and Advanced Studies, Chennai, Tamil Nadu, India

##### **Correspondence:** Afroz Patan (afroz.sps@velsuniv.ac.in)

*BMC Proceedings 2024*, **18(9):**O39

**Background**: One of the leading global causes of illness and mortality is cancer. The unchecked expansion of aberrant cells within the body is known as cancer. The most common gynaecologic malignancy among women worldwide is ovarian cancer. The recommended course of treatment for newly discovered advanced epithelial ovarian cancer is systemic platinum-taxane combination chemotherapy along with surgical cytoreduction.

**Materials and methods**: An oral, highly particular PARP-1 & PARP-2 inhibitor called niraparib has been licensed for use as adjuvant therapy in people with repetitive ovarian cancer who have shown favourable reactions to platinum-based chemotherapy. Niraparib is an extremely small-sized inhibitor of poly ADP ribose polymerase 1&2 (PARP inhibitors) that is used to treat adult patients with ovarian cancer. In individuals who are only partially responding to platinum-based chemotherapy, it is also used to cure primary fallopian tube cancer and peritoneal cancer.

**Results**: Both victims with BRCA-mutated malignancies and those without BRCA mutations have demonstrated the efficacy of niraparib. Niraparib kills cells by causing many irreversible double-stranded breaks with the Breast Cancer Susceptibility Protein (BRCA) type 1&2 alterations. Uninfected cells don’t duplicate DNA as frequently as cancer cells do, so patients are able to beat cancer.

**Conclusion:** Niraparib side effects include constipation, fatigue, and vomiting, lowered platelet as well as lowered neutrophil count. Because side effects are typically predictable in terms of their onset, duration, and degree, there is no association between the presence of side effects and niraparib’s efficacy.

## O40 A review on analytical method development and validation of estimated Capmatinib

### Harishini Sriram, Gandhimathi Retnasamy

#### Department of Pharmaceutical Chemistry and Analysis, School of Pharmaceutical Sciences, Vels Institute of Science, Technology and Advanced Studies, Chennai, Tamil Nadu, India

##### **Correspondence:** Gandhimathi Retnasamy (drgmathipharm2017@gmail.com)

*BMC Proceedings 2024*, **18(9):**O40

**Background:** The first FDA-approved medicine for the treatment of non-small cell lung cancer cells with particular mutations (resulting in a mesenchymal-epithelial junction, such as exon 14 MET skipping), capmatinib, is available today.

**Materials and methods**: This work categorizes a new fast, precise, and accurate analytical technique for detecting capmatinib in the majority and undefinable levels of pharmaceutical items. The provision of solutions like development benefits greatly from analytical procedures.

**Results**: The numerous analytical techniques that are most frequently used to pinpoint typical supply issues will be described in this article along with a classification scheme. For the majority of pharmaceutical medications and preparations, pharmaceutical analysis plays a distinctive function in quality assurance and internal control.

**Conclusion**: Several countries around the world have seen tremendous development in the pharmaceutical and pharmaceutical industries.

## O41 A systematic research review on controlled drug delivery system - osmotic drug delivery system

### Theetchanya Sankaran, Vaheeda Rehman, Shanmugasundaram Palani

#### School of Pharmaceutical Sciences, Vels Institute of Science, Technology and Advanced Sciences, Chennai, Tamil Nadu, India

##### **Correspondence:** Theetchanya Sankaran (theetchanyasankaran04@gmail.com)

*BMC Proceedings 2024*, **18(9):**O41

**Background**: Drugs are known to be released immediately by conventional drug delivery systems, making it impossible to control the release of the drug and to keep an effective concentration at the target site for an extended period. Drug release can be spatially controlled with the use of controlled drug delivery systems. The most promising technologies for regulated drug administration are osmotic pumps. Both oral delivery and implantable utilise these technologies.

**Materials and methods:** Osmotic pumps have an inner core with a semipermeable membrane coating that houses osmogens and medicines. As the volume of the core rises as a result of the water absorption, the medication solution is driven out through the delivery ports. Osmotic pumps release drugs at a pace independent of the hydrodynamics and pH of the dissolving solution. The development of the push-pull system, the Rose-Nelson pump, the Higuchi-Leeper pump, the Alzet and Osmet system, as well as the basic osmotic pump are all part of the history of osmotic systems.

**Results:** Osmotic drug delivery systems ensure precise, controlled drug release, improving patient compliance and reducing side effects by maintaining consistent drug levels in the body over an extended period.

**Conclusion:** To get around the drawbacks of conventional dosage forms, modified versions of such forms have been produced. Known as controlled-release medication delivery systems, the updated versions. Osmotic pumps are a common choice and are used to regulate medication delivery release among the many controlled-release systems. The osmotic pressure principle underlies this system, as is covered in this systematic review.

## O42 A case of small bowel neuroendocrine tumour disguised as an ileocecal intussusception

### Janani Sai Ganapathy, Govardhanan Krishnaswamy, Ravi

#### Meenakshi Medical College Hospital and Research Institute, Kanchipuram, Tamil Nadu, India

##### **Correspondence:** Janani Sai Ganapathy (jananisaiganapathy@gmail.com)

*BMC Proceedings 2024*, **18(9):**O42

**Background**: Neuroendocrine tumors (NETs) previously known as carcinoid tumors occur throughout the gastrointestinal tract, most commonly in the appendix, ileum and rectum in decreasing order of frequency. They represent a rare slow-growing neoplasm of predominantly neuroendocrine origin with non-specific characteristics thereby stumping the surgeons without pointing towards a straightforward diagnosis. Most gastrointestinal NETs are <1 cm in diameter and about 2% of these are associated with metastasis. In this case report, a small bowel neuroendocrine tumor, reiterates the diagnostic riddle that NETs pose for surgeons as most cases are diagnosed post operatively with pathological confirmation backing the diagnosis.

**Materials and methods**: A 50-year-old male presented to the casualty department with abdominal pain, vomiting, and inability to tolerate solid and liquid diet suggesting intestinal obstruction. This was radiologically proved with a CECT abdomen, which showed an ileocecal intussusception with terminal ileal wall thickening and a calcified mesenteric node mass. The patient undergoes an emergency laparotomy. With intraoperative findings showing an ileocecal mass and a calcified mesenteric node, the procedure opted for was right hemicolectomy with ileostomy and mucous fistula. Informed consent has been obtained from the patient.

**Results**: Post-operatively the resected bowel with mass was sent for histopathological examination where the pathologists determined and reported the mass to be a G1 well-differentiated neuroendocrine tumour with pathological staging of pT4N1Mx. Following surgery, the patient was followed up with chemotherapy with Capecitabine and 5 Fluorouracil.

**Conclusion**: NETs have the best prognosis of all gastrointestinal malignancies whether the disease is localized or metastatic. Surgical resection of a NET localized to its primary site approaches a 100% survival rate. Patients with metastatic disease are treated with somatostatin analogs and in case of disease progression despite somatostatin analogs, Peptide receptor radionucleotide therapy is initiated. Patients with both localized and metastatic disease are on long-term surveillance of up to 10 years as the disease is slow-growing.

## O43 Unlocking the power: crafting, analyzing, and unveiling the anticancer potential of mucuna pruriens seeds hydroalcoholic extract

### Sabbathyan Balla, Sumithra Mani

#### Department of Pharmaceutical Chemistry and Analysis, School of Pharmaceutical Sciences, Vels Institute of Science, Technology and Advanced Sciences, Chennai, Tamil Nadu, India

##### **Correspondence:** Sabbathyan Balla (sabbathyanballa@gmail.com)

*BMC Proceedings 2024*, **18(9):**O43

**Background**: Cancer, a leading global cause of death with over 277 types, presents treatment challenges due to multi-drug resistance and cytotoxicity in conventional therapies. Nanoparticles (NPs) offer promise in cancer treatment, boasting advantages like biocompatibility, precise targeting, and reduced toxicity.

**Materials and methods**: Mucuna pruriens seeds were hydroalcoholically extracted, revealing 42 bioactive compounds, including naringenin. Naringenin was then incorporated into platinum nanoparticles (PtNPs) using a green synthesis approach.

**Results**: Formulation F4, containing 100 mg naringenin and 2.0 mM chloroplatinic acid, was optimized, yielding 16 nm PtNPs. In vitro assays confirmed its anti-cancer activity, while in vivo experiments on mice demonstrated safety and efficacy against liver carcinoma.

**Conclusion**: Naringenin-loaded PtNPs hold promise as a safe and effective anti-cancer agent, providing new avenues for cancer therapy.

## O44 Review on determination of concentration of venetoclax using analytical technique

### Surya Suresh, Sumithra Mani

#### Department Pharmaceutical Chemistry and Analysis, School of Pharmaceutical Sciences, Vels Institute of Science, Technology and Advance studies, Chennai, Tamil Nadu, India

##### **Correspondence:** Sumithra Mani (sumithra.sps@velsuniv.ac.in)

*BMC Proceedings 2024*, **18(9):**O44

**Background**: Venetoclax has emerged as a breakthrough for the treatment of leukemia with a wide interindividual variability in pharmacokinetics. Herein, a rapid, sensitive, and reliable UPLC-MS/MS method for quantification of venetoclax in plasma was developed and validated.

**Materials and methods**: The method was operated in the multiple-reaction monitoring (MRM) mode to detect venetoclax at m/z transition 868.5 > 321.0 and IS at 875.5 > 321.0, respectively. Protein precipitation prior to injection into the LC-MS/MS and the analyte was separated on an ACQUITY UPLC BEH C18 column by gradient elution with acetonitrile and 0.1% formic acid in water. Linear calibration curves were obtained in the range of 25–8000 ng/mL. The specificity, recovery, matrix effect, and stability also met the acceptance criteria of FDA guidance. The method was successfully applied to analyze plasma in acute myeloid leukemia (AML) patients.

**Results**: The peak plasma concentration (C_max_) of venetoclax in Chinese AML patients was 2966.0 ± 1595.0 ng/mL while the trough concentration (C_min_) was 1018.0 ± 729.4 ng/mL. Additionally, C_max_ and C_min_ showed a positive correlation with AST levels. Furthermore, C_max_ was significantly higher in the older patients.

**Conclusion**: The present method can be applied for type 2 diabetes of venetoclax in the treatment of hematological cancers.

## O45 A high-grade malignant tumour in a paediatric proximal humerus- conundrums involved in the management- a case report

### Sriman Narayan Madan Mohan, Tarun Prashanth, Thirunthaiyan Manickam Ramachandran, Dorai Kumar Raja

#### Sri Ramachandra Institute of Higher Education and Research, Chennai, Tamil Nadu, India

##### **Correspondence:** Sriman Narayan Madan Mohan (sriman.gunner06@gmail.com)

*BMC Proceedings 2024*, **18(9):**O45

**Background:** Osteosarcoma is the most common primary malignant bone tumor affecting the long bones of all age groups. The following is a case report of an adolescent diagnosed with the same, treated with neoadjuvant chemotherapy followed by surgical resection and custom mega prosthesis application.

**Materials and methods:** A 12-year-old girl presented with right arm pain and swelling progressively increasing in size. She was clinically and radiologically diagnosed with Proximal Humerus mixed lytic sclerotic lesion which was confirmed to be a high-grade Osteoblastic variant of Osteosarcoma of Right Proximal Humerus on CT-guided biopsy with features suggestive of Osteosarcoma NOS-Osteoblastic variant, with no active secondaries on PET-CT. She underwent neoadjuvant chemotherapy, followed by surgical excision of mass and reconstruction of the proximal humerus using a custom mega prosthesis. Informed consent has been obtained from the guardian of the patient.

**Results:** The patient underwent two cycles of chemotherapy, followed by surgical excision of the proximal humerus mass with a 4cm wide safe margin, and reconstruction using custom mega endoprosthesis. Soft Tissue reconstruction was done with Mesh and Suture anchors. The patient was given postoperative rehabilitation and continued on neoadjuvant chemotherapy cycles. During the follow-up period, 3 months post-surgery, the patient resumed going to school, and started activities like drawing and writing with her right hand.

**Conclusion:** Following clinical evaluation, MRI plays a significant role in describing the lesion’s extent and plan for surgical management. Histopathological examination of the tumor from intra-operative resection was graded-ypT1 ypN, high-grade osteosarcoma with margins free of tumor. For high-grade Osteogenic Sarcoma, a combination of surgical resection along with multiagent chemotherapy is crucial. Treatment options include Limb Salvage Surgery-wide tumor resection and proximal humerus reconstruction with Custom Mega prosthesis, allograft, allograft-prosthesis composite with shoulder arthroplasty, reverse shoulder hemiarthroplasty and postoperative chemotherapy with concurrent rehabilitation is vital.

## O46 Teaching-learning experiences in Labster: the allied medical degree programs in focus

### Adelle N. Sanchez

#### University of Baguio, Baguio, Philippines

##### **Correspondence:** Adelle N. Sanchez (adellesanchez@e.ubaguio.edu)

*BMC Proceedings 2024*, **18(9):**O46

**Background:** The high fidelity of the Labster laboratory simulation has been a good tool for conducting laboratory courses online. It simulates a real laboratory setting complete with equipment and procedures. This feature enables learners to experience the real-world laboratory set-up which will be useful in their hybrid flexible learning and face-to-face learning. The use of this simulation enables learners to achieve the cognitive, affective, psychomotor, and laboratory skills needed in the Biochemistry course.

**Materials and methods:** A retrospective one-shot case study and phenomenological research design was used to investigate the impact of Labster on the Allied Medical Degree Programs of the University of Baguio. The participants included 95 students from Medical Laboratory Science, Physical Therapy, Dentistry, and Nursing students as well as 3 Biochemistry instructors.

**Results:** Results showed that most of the skills are enhanced with the introduction and use of the simulation in online classes such as affective, psychomotor and laboratory skills. However, the respondents had low cognitive skills. Some challenges were encountered in the use of the simulation such as the needed specs for gadgets to be able to run the simulation, limited internet connectivity, use of the simulation, and inability for the cartridges to be downloaded so that it would be incorporated into the learning management system. Instructors addressed the impediments in the use of the simulation by allowing learners to view videos that are close to Labster and are thoroughly discussed during synchronous classes.

**Conclusion:** Labster laboratory simulation is a good tool in online laboratory classes however, it should also be tweaked into user and gadget-friendly applications so that learners from progressing countries can make use of it.

## O47 The neck posture practices among University of Baguio BSPT and BSMLS students during distance learning

### Hazel Mang-osan, Crystal Agbalog, Krystal Balabbo, Kyra Battuing, Nadine Gomez, Jeffrey Salonga

#### University of Baguio, Baguio, Philippines

##### **Correspondence:** Hazel Mang-osan (20190891@s.ubaguio.edu)

*BMC Proceedings 2024*, **18(9):**O47

**Background**: The implementation of quarantine protocols caused all educational activities to move to online platforms. Consequently, complaints of musculoskeletal disorders such as neck pain have been rising due to the continuous use of electronic gadgets throughout the day. This study aimed to determine the extent of neck posture practices among the University of Baguio BSPT and BSMLS students during distance learning; and determine the significant difference in the extent of neck posture practices according to sex, duration of class, and types of gadgets used.

**Materials and methods:** A quantitative descriptive design was utilised. A total of 219 students participated using an online self-administered questionnaire. Weighted mean was used to determine the general neck posture practices, ANOVA to determine significant differences between the duration of the class and the type of gadgets, and t-test to examine the differences between male and female neck posture practices.

**Results:** Results showed that the respondents rarely practice the neck posture practices. T-tests for the two sexes revealed that the extent of neck posture practices of 186 female participants compared to 33 male participants demonstrated a non-significant difference. Findings in the ANOVA showed that there was also no significant difference in the duration of classes and the type of gadget used.

**Conclusion:** The results of the study demonstrate that students rarely perform neck posture practices despite being aware of the proper ergonomics. Regardless of the sex, duration of the class, and type of gadgets used by the students, the majority are prone to musculoskeletal discomforts as a result of the infrequent practice of proper neck postures.

## O48 An overview of computer system validation

### Seema Thiruvasagam, Afroz Patan

#### Department Pharmaceutical Chemistry and Analysis, School of Pharmaceutical Sciences, Vels Institute of Science, Technology and Advance Studies, Chennai, Tamil Nadu, India

##### **Correspondence:** Afroz Patan (afroz.sps@velsuniv.ac.in)

*BMC Proceedings 2024*, **18(9):**O48

**Background**: Computer system validation (CSV) is a critical process for ensuring the quality and reliability of computerized systems in regulated industries such as pharmaceuticals, medical devices, and biotechnology.

**Materials and methods:** To ensure compliance with rules like 21 CFR Part 11, this procedure is mandated by regulatory agencies like the FDA. The specifications for electronic documents & signatures used in industries under FDA regulation are outlined in 21 CFR Part 11. It covers every electronic document that is produced, changed, kept up to date, archived, retrieved, or sent, as well as every electronic signature used to sign such documents.

**Results**: The 21 CFR Part 11 regulations and compliance guiding principles, as well as the ideas of computer system validation, will be introduced in this overview. Also, it will go through the various stages of the CSV process, such as planning, design, testing, and maintenance as well as the significance of risk management and all documentation during the procedure.

**Conclusion**: The overview will also discuss how automation & software tools are used in CSV and will highlight some of the typical issues as well as best practices related to this procedure.

## O49 Academic and physical therapy licensure exam performance: an evaluation

### Kimwell S. Anague, Ruth D. Fernandez

#### University of Baguio, Baguio, Philippines

##### **Correspondence:** Kimwell S. Anague (ksanague@e.ubaguio.edu)

*BMC Proceedings 2024*, **18(9):**O49

**Background:** A measure of success of curriculum implementation is best manifested in a graduate’s academic performance. The study evaluated the level of academic performance of BSPT students and their Physical Therapy (P.T.) licensure examination performance. It determined the relationship between academic and P.T. licensure performances in the lecture and laboratory components.

**Materials and methods:** A quantitative descriptive study that utilised the ex-post facto design was used. It included academic records of all UB BSPT graduates who have taken the Philippine Physical Therapy Licensure Examinations from August 2015 to August 2019. Total enumeration was used.

**Results:** Results showed that BSPT students have a fair academic performance with APK lecture and M.S. laboratory and average academic performance with the other components. There are strong and significant relationships between the academic performance of BSPT students in the Lecture and Laboratory components of the BSPT professional subjects. There are significant differences in the P.T. licensure performance in APK and P.T. App components among the batches of BSPT graduates. Academic performance significantly predicts performance in the P.T. licensure exams regardless of academic subject clusters in APK, MS, and P.T. App.

**Conclusion:** Students of the University of Baguio, Bachelor of Science in Physical Therapy place preference on experiential learning approaches and perform better in laboratory components. Recommendations include providing balanced learning experiences for both theoretical and practical components of BSPT courses, reviewing the grading system, and strict implementation of the school’s retention policy.

## O50 Effects of recreational cycling on low back pain among college students of the University of Baguio

### Gene Zionore Baysa-Pee, Amani Gaminde Khaled Galal Hussein, Asma Gaminde, Khaled Galal Hussein, Jaira Gwen Ramos, Jonaira Daway Ronquillo, Kimwell Anague

#### University of Baguio, Baguio, Philippines

##### **Correspondence:** Gene Zionore Baysa-Pee (20201197@s.ubaguio.edu)

*BMC Proceedings 2024*, **18(9):**O50

**Background**: Cycling as a recreational activity may come with risks, as it causes prolonged lumbar flexion, which has been linked to low back pain (LBP). The study investigated the effects of recreational cycling among college students at the University of Baguio.

**Materials and methods:** Simple random sampling was used to recruit 130 cyclists from the university. Respondents completed a questionnaire where they reported their weekly cycling characteristics. The questionnaire also served to profile respondents.

**Results:** Most of the respondents were female, lacked a history of LBP, performed recreational cycling twice a week or less, cycled for less than 2 hours per session, and had been doing recreational cycling for less than one year. Furthermore, the respondents usually cycled on flat terrain, utilizing upper handlebar types. The Chi-square test for independence revealed no significant associations between LBP and the moderator variables sex, frequency of cycling, duration of a single cycling session, history of LBP, and handlebar type.

**Conclusion:** Recreational cycling has no significant effect on LBP and would be considered safe for most people. The findings of this study suggest that cycling, as done within the parameters defined in this study, will likely not aggravate LBP. The researchers would even go on to say that cycling performed this way is a potentially beneficial exercise for LBP. This is because while cycling as an exercise places stress on the lower back, taking adequate amounts of rest in between cycling sessions allows the strengthening of lumbar muscles while avoiding injury.

## P1 Alpha (α)-mangostin (*Garcinia mangostana* L.): a natural anti-inflammatory agent in diabetic wound management

### Melonney Patrick¹, Wan Najwa Wan Mohd Zohdi², Suhaila Abd.Muid^1,4^, Effat Omar^3,4^

#### ^1^Department of Biochemistry & Molecular Medicine, Faculty of Medicine, Universiti Teknologi MARA Sungai Buloh, Selangor, Malaysia; ^2^Department of Rehabilitation Medicine, Faculty of Medicine, Universiti Teknologi MARA Sungai Buloh, Selangor, Malaysia; ^3^Department of Pathology, Faculty of Medicine, Universiti Teknologi MARA Sungai Buloh, Selangor, Malaysia; ^4^Institute of Pathology, Laboratory and Forensic Medicine (I-PPerForM), Universiti Teknologi MARA Sungai Buloh, Selangor, Malaysia

##### **Correspondence:** Melonney Patrick (melnymell@yahoo.com)

*BMC Proceedings 2024*, **18(9):**P1

**Background**: Diabetic wounds present a unique challenge in medical treatment due to their slower healing process than normal wounds. Macrophages play a crucial role in all phases of normal wound healing, including inflammation, proliferation, and remodelling. In cases of impaired wound healing, macrophages can disrupt the balance between tissue inhibitors of metalloproteinase (TIMPs) and matrix metalloproteinase (MMPs) while also suppressing inflammation through the upregulation of interleukin-6 (*IL-6*). Alpha-mangostin, a natural xanthone derived from the pericarp of the mangosteen, has gained considerable attention due to its anti-inflammatory, suggesting its potential to promote wound healing, particularly diabetic foot ulcers, and remains an enigma. Alpha-mangostin might expedite the healing process in diabetic wounds remains unclear. To investigate the potential effects of alpha-mangostin on diabetic wound healing by evaluating its impact on *IL-6, MMP-9*, and *TIMP-2* secretion in macrophage cells.

**Materials and methods**: Human monocytic cell line (THP-1) cells were incubated with a 35 mM glucose solution for 72 hours to create a glucose-enriched medium. The cells were then incubated with alpha**-**mangostin (0.15, 2.5, and 5 μg/ml), positive control (carboxymethyl cellulose, and negative controls (high glucose and culture medium alone). Protein expression and gene expression (*IL-6, TIMP-2*, and *MMP-9*) were measured using ELISA and qPCR.

**Results**: Alpha**-**mangostin (0.15 and 2.5 μg/ml) reduced *MMP-9* and *IL-6* secretion levels compared to glucose controls on both protein and gene expression. There was no significant increase in *TIMP-2* protein levels across any of the treatment groups compared to glucose control. However, a notable increase was observed in gene expression with a concentration of 2.5 μg/ml.

**Conclusion**: Alpha-mangostin lowered *MMP-9* and *IL-6* secretion in both protein and gene expression and increased *TIMP-2* in gene expression, suggesting potential use in diabetic wound healing.

## P2 Level of knowledge and causes on gastritis among high school students

### Luqman Bin Saidin, Nurelveen Fitri Binti Hisham, Nurul Ashifa Binti Zainol, Muhammad Naqiuddin Hakim Bin Alias, Iman Zul Hakim Bin Amran Alimin, Muhammad Asyraf Salleh

#### Department of Medical Sciences, Faculty of Health Sciences, University College of MAIWP International, Kuala Lumpur, Malaysia

##### **Correspondence:** Muhammad Asyraf Salleh (asyraf@ucmi.edu.my)

*BMC Proceedings 2024*, **18(9):**P2

**Background**: Gastritis is an inflammatory condition of the gastric mucosa that exhibits changes related to etiology and host response. Students suffer from gastritis because of stress, lack of gastritis information, less time to eat and improper eating patterns. This study aims to determine knowledge and causes of gastritis among high school students.

**Materials and methods:** In this study, we explore the knowledge among high school students who clinically diagnosis have gastric. We recruited a cross-sectional study and the survey collected data on sociodemographic and self-administrated structured questionnaires with close questions. Descriptive statistical and t-test analysis was used using SPSS version 26.

**Results:** A total of 100 respondents were involved, and the mean of knowledge is 2.92 ± 0.26 where 97% of the respondents have a high level while another 3% have a low level of knowledge on gastritis. Our results show that skipping breakfast (51.35%) is the main cause of gastritis followed by frequently eating spicy food (33.43%) and eating sour food (16.22%). These findings also show that there is no significant difference in the level of knowledge between genders.

**Conclusion:** In conclusion, this study found that most of the gastritis high school students have a high level of knowledge. Skipping breakfast is the main cause and schools need to provide urgent intervention strategies to avoid uncertainty problems among these students.

## P3 Renal transplant: knowledge among secondary school students in Selangor

### Muhammad Amir Hafiz Bin Khairuddin, Nur Atiqah Binti Anuar, Ainur Aelisa Binti Baharuddin, Puteri Noorzita Khan Binti Zainal Abidin, Muhamad Zakuan Abdullah

#### Department of Medical Sciences, Faculty of Health Sciences, University College of MAIWP International, Kuala Lumpur, Malaysia

##### **Correspondence:** Muhamad Zakuan Abdullah (m.zakuan@ucmi.edu.my)

*BMC Proceedings 2024*, **18(9):**P3

**Background**: A renal transplant is a renal therapy that involves transplanting a damaged renal from an End Stage Renal Disease (ESRD) recipient and replacing it with a new renal from a donor. Although this organ transplant is useful and very helpful, many ESRD patients are unable to undergo an organ transplant within the specified time and need to receive long-term dialysis treatment. However, the lack of pledgers or donors made the situation difficult and the mortality rate increased. Lack of exposure and discussion among young people about this issue contribute to their unwillingness toward organ donation. So, this research is focused on determining the level of knowledge among secondary school students about renal transplants. The research also aimed to compare the level of renal transplant knowledge based on gender.

**Materials and methods:** A 21-item self-administered questionnaire was used. It assessed the level of knowledge regarding renal transplant using the Likert rating scale. It also asked for demographic data. Of the 100 respondents who participated, 50 (50%) were boys while 50 (50%) of them were girls.

**Results:** From this study, the majority of them (52%) had a high level of knowledge about renal transplant whereas 48% of students had a low level. When compared by gender using the t-Test, there was a statistically significant difference among males and females toward renal transplant (*p* = 0.009).

**Conclusion:** Most of those students have heard about transplants. However, information on kidney transplants has to be spread out to increase public knowledge and positive attitudes to support renal transplant programs to reduce the number of cases of ESRD.

## P4 The level of knowledge and symptom of Polycystic Ovary Syndrome (PCOS) among women in Klang

### Bakhita Stephanie Ong Swee Lian, Nazaratul Nazira Nazib, Nik Nuranis Syafiqah Nik Mohd Sabri, Roslinda Saiful Salleh, Muhammad Haziq Mohamat Suhaim, Zulfa Zakuan

#### Department of Medical Sciences, Faculty of Health Sciences, University College of MAIWP International, Kuala Lumpur, Malaysia

##### **Correspondence:** Zulfa Zakuan (zulfa@ucmi.edu.my)

*BMC Proceedings 2024*, **18(9):**P4

**Background:** Polycystic Ovarian Syndrome (PCOS) is the most common endocrine disorder among women between the ages of 18 to 44 years. PCOS cannot be prevented but early diagnosis and treatment help prevent long-term complications. This study aimed to determine the level of knowledge about PCOS and measure the severity of symptoms of PCOS and lifestyle among women in Klang.

**Materials and methods:** In this cross-sectional study, data was collected through questionnaires administered to women respondents in Klang, Selangor. The respondents were selected through quota sampling. The data was collected through self-administered structured questionnaires related to knowledge, severity of symptoms of PCOS experienced by the respondents, and their lifestyle. Descriptive and correlation statistics were used for data analysis using SPSS version 26.

**Results:** A total of 95 respondents participated in the study. 97.9% of the respondents showed a high level of knowledge. Meanwhile, participants were shown to have severe PCOS symptoms (2.743 ± 0.275) with poor lifestyle (2.704 ± 0.392). Lastly, there is a significant correlation between lifestyle and symptoms of PCOS (*p*< .001).

**Conclusion:** In conclusion, the study has shown that most respondents have a high level of knowledge, severe PCOS symptoms and poor lifestyle. Symptoms and lifestyle showed a positive and significant correlation. In-depth studies are needed to discover the factors causing the rise of PCOS cases among women.

## P5 Water quality impacts of *Hypostomus plecostomus* towards *Danio rerio* tank system

### Siti Sarah Mohd Asri, Rushduddin Al Jufri Roosli

#### Department of Medical Sciences, Faculty of Health Sciences, University College of MAIWP International, Kuala Lumpur, Malaysia

##### **Correspondence:** Rushduddin Al Jufri Roosli (jufri@ucmi.edu.my)

*BMC Proceedings 2024*, **18(9):**P5

**Background:** *Hypostomus plecostomus* (pleco) cleans aquatic tanks by consuming algae and uneaten food, and it is usually kept in systems with the other fishes. However, no studies have been done to show how pleco impacts the water quality of tank systems. *Danio rerio* (zebrafish) tank system maintenance involves an emphasis on several factors, including temperature, illumination, water quality, and pH. Thus, the purpose of this study is to determine whether having pleco improves the water quality of the zebrafish tank system.

**Materials and methods:** *Hypostomus plecostomus* between the ages of 6 and 9 months and Danio rerio between the ages of 2 and 3 months were used. 30 zebrafish were grouped into 3 different tanks with 10 zebrafish and 0, 1 or 2 number of plecos respectively in each tank. The tanks were observed daily for two weeks and water parameters such as ammonia, nitrite, nitrate, pH, temperature, total dissolved solute (TDS), electrical conductivity (EC) and dissolved oxygen (DO) were recorded.

**Results:** The results showed that the parameters of water quality in the tanks with plecos are better compared to the tanks without plecos. The tank with 2 plecos was also found to have a better reading compared to the tank with only 1 pleco.

**Conclusion:** These early findings indicate that the existence of pleco indeed improves the water quality of the tank system. However, further investigation is going on to conduct triplicate experiments for longer periods and with a more varying number of plecos.

**Acknowledgement:** This study was funded by Tun Ahmad Sarji Research Endowment Fund (TASREF) [UCMI/TNCPP/RMC/Gearan/2022 (51)] from University College of MAIWP International.

## P6 Toxicity of *Physalis angulata* L. on zebrafish embryos

### Ain Munirah Muhamad Sofian, Zulfa Zakuan

#### Department of Medical Sciences, Faculty of Health Sciences, University College of MAIWP International, Kuala Lumpur, Malaysia

##### **Correspondence:** Zulfa Zakuan (zulfa@ucmi.edu.my)

*BMC Proceedings 2024*, **18(9):**P6

**Background:** *Physalis angulata* L. which is commonly known as cutleaf groundcherry or letup-letup is famously known for its medicinal properties for wound treatment and healing. Several studies have shown that the bioactive compounds from *P. angulata* L. possessed biological activity such as antiasthmatic, antimalarial and antiallergic. Previous studies have also shown that the extract of *P. angulata* L. was used to test on post-larvae of tiger shrimp (*Penaeus monodon*). However, to date, no research has been done on the toxicity of *P. angulata* L. with zebrafish embryos. Therefore, this research is to assess the toxicity of *P. angulata* L. ethanolic extract on zebrafish embryos.

**Materials and methods:** The *P. angulata* L. leaves were dried and grounded before it was soaked in 70% ethanol. Using the maceration method, the ethanolic extract of *P. angulata* L. was extracted. For the embryo toxicity test, twenty fertilised embryos were exposed to different concentrations of ethanolic *P. angulata* L. extract (0, 100, 250, 500, 750 and 1000 mg/ml). The mortality of the embryo was assessed by the coagulation of the embryo and the death of larvae. The lethality concentrations (LC50) were determined at different hours of post-fertilization (hpf). The positive control used was 3,4-dichloroaniline, while the negative control was E3 medium.

**Results:** Generally, increasing concentrations of the extract showed an increase in mortality of the embryo. The Lc50 for 24 hpf, 48 hpf, and 72 hpf were 1981 mg/ml, 540 mg/ml and 226 mg/ml respectively. Some of the hatched larvae showed apparent deformities due to the exposure.

**Conclusion:** *P. angulata* L. showed low toxicity to the embryo of zebrafish. Detailed assessment of the developmental points of the embryo and larvae is needed to determine its potential teratogenicity.

## P7 Prevalence of COVID-19 status during pandemic among Malaysian population

### Rudyanova Arifin^1^, Haily Liduin Koyou^1^, Vasudevan Ramachandran^1^, Adibah Syahnaz Zahari^2^

#### ^1^Department of Medical Sciences, Faculty of Health Sciences, University College of MAIWP International, Kuala Lumpur, Malaysia; ^2^RADILAB Diagnostics Sdn. Bhd, Bukit Jalil, Kuala Lumpur, Malaysia

##### **Correspondence:** Haily Liduin Koyou (haily@ucmi.edu.my)

*BMC Proceedings 2024*, **18(9):**P7

**Background:** Coronavirus disease (COVID-19) is an infectious disease caused by the SARS-CoV-2 virus which emerged at the end of 2019 in Wuhan, China. COVID-19 has resulted in the death of millions of people worldwide including the Malaysian population. 7 waves of COVID-19 occurred during the pandemic in Malaysia. Studies in Malaysia that investigate the prevalence of COVID-19 for each wave are less known. This study was initiated to provide such data on COVID-19 subjects reported from a single centre.

**Materials and methods:** In this population-based cross-sectional study, 771 subjects were analysed from the records collected from RADILAB, Malaysia. All their useful information was recorded from Malaysia’s Public Health Laboratory Information System (SIMKA). A complete tabulation of their demographic factors was included together with statistical analysis regarding percentages, standard deviation, and mean values.

**Results:** In total, 425 (55%) are males and 346 (45%) are females in which 264 of them were infected with COVID-19. Among 264 infected cases, 133 (50%) males and 131 (50%) females were infected. The recurrence rate shows that 753 (97.7%) of them had never experienced a recurrence of COVID-19 while 18 (2.3%) of them experienced only one-time recurrence. The age group of 31-40 years old recorded the highest positive cases (87 (33%)). Chinese ethnic recorded the highest number of positive cases (157 (59%)) compared to other ethnicities. Lastly, the highest positive cases of 150 people were recorded during the 5th wave.

**Conclusion:** Further analysis is needed regarding clinical manifestations, vaccination status and the severity of this disease to determine the effect of COVID-19 during the pandemic in Malaysia.

**Acknowledgement**

This study has been fully supported by Tun Ahmad Sarji Endowment Fund (TASREF) UCMI/TNCPP/RMC/Geran/2024 (63), University College of MAIWP International, Kuala Lumpur, Malaysia.

## P8 Analysis of *CCR2* gene polymorphism among stage 5 ESRD subjects

### Nur Ameera Asyiqin Shamsuri, Muhammad Asyraf Salleh, Vasudevan Ramachandran

#### Department of Medical Sciences, Faculty of Health Sciences, University College of MAIWP International, Kuala Lumpur, Malaysia

##### **Correspondence:** Muhammad Asyraf Salleh (asyraf@ucmi.edu.my)

*BMC Proceedings 2024*, **18(9):**P8

**Background:** The prevalence of chronic kidney disease (CKD) in Malaysia is 9.07% of the total population, with 0.36% having stage 5 CKD or end-stage renal disease (ESRD). Several studies have reported that *CCR2* plasma levels were significantly higher in patients with ESRD. The present study aims to determine the frequency of the genotype and compare the genotype by age, weight and eGFR value.

**Materials and methods:** This experimental study was conducted at the MAIWP-UCMI Hemodialysis Centre in Batu Muda and Jalan Pahang branch. A total of 28 ESRD patients were treated with hemodialysis, were interviewed and signed informed consent. DNA was extracted from secondary blood samples taken from the ESRD patient. The *CCR2* genotype (II, ID and DD) was determined by polymerase chain reaction (PCR) analysis.

**Results:** The sample of 28 patients comprises 10 males (35.7%) and 18 females (64.3%). Among the three genotypes, the II genotype was the highest (19 patients, 67.9%), followed by the ID genotype (7 patients, 25%) and DD genotype (2 patients, 7.1%). *CCR2* showed no association with ESRD in comparison with age (*p* = 0.493), weight (*p* = 0.772) and *eGFR* value (*p* = 0.761) using the Chi-square test.

**Conclusion:** The results of this study are the outcomes of the comparison between genotype and patient’s age, weight and *eGFR* value. However, the data presented uses a small sample size and the statistical analyses show no significant difference. Therefore, further studies are suggested using a larger sample size for clearer results to aid in the development of epidemiological studies in Malaysia.

## P9 Effect of hemodialysis on blood morphology in patients with end-stage renal disease at MAIWP Hemodialysis Center, Batu Muda, Malaysia

### Raihana Amani Binti A Razak, Henkie Isahwan Ahmad Mulyadi Lai

#### Department of Medical Sciences, Faculty of Health Sciences, University College of MAIWP International, Kuala Lumpur, Malaysia

##### **Correspondence:** Henkie Isahwan Ahmad Mulyadi Lai (henkie@ucmi.edu.my)

*BMC Proceedings 2024*, **18(9):**P9

**Background**: End-stage renal disease (ESRD) is the most severe form of kidney failure, necessitating dialysis or a transplant for survival. The prevalence of ESRD has been increasing. Hemodialysis is a process in which blood is filtered using a dialysis machine and a specific filter known as an artificial kidney or dialyzer. There is limited data available in our community regarding the impact of hemodialysis on hematological parameters, particularly blood morphology. The objective of this study is to determine the effect of hemodialysis on blood morphology.

**Materials and methods**: This analytical study is conducted at the MAIWP Hemodialysis Center in Batu Muda, focusing on ESRD patients. A total of 100 blood samples will be collected from 50 hemodialysis patients, both before and after dialysis. Blood samples will be taken from hemodialysis patients to create peripheral blood films (PBF), which will be observed under a microscope to assess blood morphology. Various types of red blood cell (RBC) morphology will be identified among hemodialysis patients at MAIWP Hemodialysis Center.

**Results:** A total of 100 blood cells were examined before and after dialysis from 50 hemodialysis patients. Overall, 92% of ovalocyte-shaped cells were observed, 87% were spherocytes and ovalocytes, and 2% were ellipsocytes.

**Conclusion**: These findings suggest that hemodialysis patients are more likely to have ovalocytes, spherocytes, and ovalocytes compared to control patients.

## P10 Analysis of *CCR5* gene polymorphism among stage 5 ESRD subjects

### Nurul Izzah Yusof, Nurul Ain Abu Bakar, Vasudevan Ramachandran

#### Department of Medical Sciences, Faculty of Health Sciences, University College of MAIWP International, Kuala Lumpur, Malaysia

##### **Correspondence:** Nurul Ain Abu Bakar (nurul_ain@ucmi.edu.my)

*BMC Proceedings 2024*, **18(9):**P10

**Background:** In Malaysia, 58% of ESRD patients are diabetic. Findings from a population-based cross-sectional study in 2020 reported that the prevalence of chronic kidney disease (CKD) in Malaysia had increased by 6.41%, rising from 9.07% to 15.48%, with stage 5 CKD or end-stage renal disease (ESRD). The highest percentage of dialysis patients was found in the 55-64 age group. This study aims to determine the genotype comparison between age, weight, *eGFR* value, and the frequency of genotypes.

**Materials and methods:** This study was conducted at the MAIWP-UCMI Hemodialysis Centre in Jalan Pahang and the Batu Muda branch. Informed consent was obtained from all the recruited patients prior to the study. A total of 28 patients received treatment at the hemodialysis centre. DNA was extracted from blood samples taken from the ESRD patients, and the *CCR5* genotype (II, ID, and DD) was determined using polymerase chain reaction (PCR).

**Results:** Out of the 28 patients, there were 18 female patients (64.3%) and 10 male patients (35.7%). Among the three genotypic categories, the II genotype was the most prevalent (11 patients, 39.3%), followed by the ID genotype (10 patients, 35.7%), and the DD genotype (7 patients, 25.0%). The analysis of *CCR5* indicated no association with ESRD when compared to age (*p* = 0.022), weight (*p* = 0.948), and *eGFR* value (*p* = 0.709).

**Conclusion:** Study findings compared genotypes with patient age, weight, and *eGFR* values. Small sample data revealed no significant differences, necessitating a larger ESRD subject sample for further investigation.

## P11 Anti-inflammatory activity of a cream combination of *Moringa oleifera* L. extract and *Zingiber officinale* R. in mice induced with carrageenan

### Intan Andini, Ajeng Dian Pertiwi, Tuhfatul Ulya, Sri Idawati

#### D3 Pharmacy, Politeknik Medica Farma Husada Mataram, Sekarbela, Indonesia

##### **Correspondence:** Intan Andini (addian90@gmail.com)

*BMC Proceedings 2024*, **18(9):**P11

**Background**: Inflammation is a normal protective response to tissue damage caused by physical, chemical, and microbiological trauma. This study aims to determine the anti-inflammatory effects of a combination cream containing Moringa leaf extract (*Moringa oleifera* L.) and ginger (*Zingiber officinale* R.) on mice (*Mus musculus*) induced with 1% carrageenan

**Materials and methods:** This study used 16 male mice divided into 4 groups. Group I was given a cream base (negative control), Group II was given 2.5% hydrocortisone (positive control), and Groups III and IV were treated with a combination cream containing Moringa leaf extract and ginger at concentrations of 5% and 10%. The cream was applied topically, and measurements of the male mice’s legs were taken every hour for a total of 5 hours.

**Results:** The average percentages of inflammatory inhibition in the positive control, creams with extract concentrations of 5%, and 10% were 35.53%, 47.29%, and 41.03%, respectively. It can be concluded that a concentration of 5% exhibits the highest percentage of inflammatory inhibition. Statistical tests indicate that the p-value for the difference in measurement hours is 0.502, which is greater than the error threshold of 5%. This suggests that the measurement time does not affect the edema volume of each treatment group.

**Conclusion:** A combination cream containing Moringa leaf extract (*Moringa oleifera* L.) and ginger (*Zingiber officinale* R.) exhibits anti-inflammatory activity in mice. Cream with a concentration of 5% demonstrates the most optimal anti-inflammatory activity.

## P12 Inhibiting streptococcus mutans biofilm formation by suppressing sucrose decomposition: exploring the potential of oral probiotic lactobacillus coagulans

### Aini

#### D3 Pharmacy, Polytechnic of Medica Farma Husada Mataram, Sekarbela, Indonesia

##### **Correspondence:** Aini (ainie.mfh@gmail.com)

*BMC Proceedings 2024*, **18(9):**P12

**Background**: Probiotics are supplements containing lactic acid bacteria, known for their ability to metabolize various sugars, including lactose. The presence of Lactobacillus coagulans demonstrates a remarkable antagonistic effect against Streptococcus mutans and offers beneficial properties as an oral probiotic. Additionally, Bacillus coagulans exhibits a safe probiotic profile, characterized by the absence of hemolytic activity, no production of D-lactate, no production of biogenic amino acids, and susceptibility to antibiotics. The aim of this study is to identify Lactobacillus coagulans, a type of lactic acid bacteria, isolated from local Lombok nira as potential probiotics.

**Materials and methods**: To evaluate the impact quantitatively, the amount of Streptococcus mutans insoluble in the EPS biofilm was measured using the anthrone method. Data are presented as mean ± standard deviation. Statistical analysis was performed using one-way ANOVA.

**Results**: The double comparison test revealed a significant difference when compared to the control (*p* < 0.05).

**Conclusion**: This finding suggests the potential use of Lactobacillus coagulans in the development of oral probiotics.

## P13 Molecular analysis of endophytic bacterial isolates from papaya leaves (*Carica papaya* L.)

### Imelda Amelia Damayanti, Dhika Juliana Sukmana, Edy Kurniawan, Baiq Isti Hijriani, Idham Halid

#### D3 Medical Laboratory Technology, Politeknik Medica Farma Husada Mataram, Sekarbela, Indonesia

##### **Correspondence:** Dhika Juliana Sukmana (dhika.juliana.dj@gmail.com)

*BMC Proceedings 2024*, **18(9):**P13

**Background**: Endophytic bacteria live within the vascular tissue of plants without causing negative effects. This study aims to identify the endophytic bacteria obtained from papaya leaf isolates through microbiological and molecular methods, using the PCR technique.

**Materials and methods:** This research was a descriptive observational study, which involved two samples of papaya leaves isolated from Batu Ringgit Street, Sekarbela, Mataram. The samples were named D5 and D7, based on specific characteristics determined by the researcher. These samples were cultured on Nutrient Agar (NA) media, followed by a Gram stain test. Colonies that were white and exhibited characteristics of endophytic bacteria on Gram staining were then purified on Sodium Agar Slant media. When the bacteria displayed the characteristics of endophytic bacteria on Gram staining, further biochemical tests and microbiological identification were performed.

**Results:** The results showed that both of the 2 isolated papaya leaf samples tested positive for endophytic bacteria, identified as *Bacillus cereus* and *Bacillus pumilus*. Subsequently, the PCR stage was carried out using the conventional PCR method to confirm the presence of endophytic bacteria in the papaya leaf samples, targeting a 1300 bp gene.

**Conclusion:** The study’s results found that the target genes were successfully identified as *Bacillus cereus* and *Bacillus pumilus*, both separated and aligned with the 1300 bp mark, through microbiological and molecular analysis.

## P14 Impact of Pecha Kucha on students’ employability skills

### Zulkifli Khair^1^, Ida Idayu Muhamad^2^, Ana Haziqah A Rashid^1^

#### ^1^School of Human Resource Development and Psychology, Faculty of Social Sciences and Humanities, Universiti Teknologi Malaysia, Johor, Malaysia; ^2^Faculty of Chemical and Energy Engineering, Universiti Teknologi Malaysia, Johor, Malaysia

##### **Correspondence:** Zulkifli Khair (zulkiflih@utm.my)

*BMC Proceedings 2024*, **18(9):**P14

**Background**: Instead of using the traditional model of PowerPoint presentation, Pecha Kucha has become an alternative model for presentation activity in the classroom. This project explored the impact of Pecha Kucha on employability skills.

**Materials and methods:** The participants were 50 students who delivered Pecha Kucha presentation as their presentation assessment. Structured and unstructured interviews were used.

**Results:** Three major codes; efficient, time management and generic skills were generated from the students.

**Conclusion:** Pecha Kucha enabled them a clearer understanding of the subject before conveying the ideas clearly and succinctly within a limited time frame, which enhances their generic skills like critical thinking skills, creative thinking skills and resilience.

## P15 Building trust in online shopping: a B40 consumer perspective in Klang Valley

### Nur Iffah Adila Mat Jib^1^, Anis Anida Shaharudin^1^, Nur Izzati Mohd Rosli^1^, Latif Raj Mohd Shamsudin^1^, Zuraini Alias^1^, Nurul Nadiah Abdul Rahman^1^, Siti Murni Mat Khairi^2^

#### ^1^Departement of Management, Faculty of Management, Education, and Humanities, University College of MAIWP International, Kuala Lumpur, Malaysia; ^2^Universiti Keusahawanan Koperasi Malaysia, Selangor, Malaysia

##### **Correspondence:** Nur Iffah Adila Mat Jib (iffahadila@ucmi.edu.my)

*BMC Proceedings 2024*, **18(9):**P15

**Background**: Trust is viewed as a fundamental and complex human interaction and relationship that involves belief or confidence in the reliability, integrity, and honesty of a person, group, organization, or system. Trust plays a significant role in e-commerce because it involves making financial transactions and sharing personal information online. Building and maintaining trust can lead to loyal customers and positive word-of-mouth, which are crucial for the long-term success of e-commerce businesses. Hence, this study comprehensively explores how B40 consumers residing in Klang Valley perceive trust in the context of online shopping, particularly concerning essential aspects such as website security, seller reputation, and the reliability of on-time delivery services.

**Materials and methods:** A sample of 138 respondents representing B40 consumer groups who have experienced online purchasing in Klang Valley participated in the questionnaire survey. Statistical Package for the Social Sciences (SPSS) was utilised to explain the relationship between website security, seller reputation, and on-time delivery towards trust in online shopping. The collected data were analysed using descriptive and inferential statistical approaches.

**Results:** The findings of the study indicate that website security, seller reputation and on-time delivery are positively related to trust in online shopping. Our results suggest that seller reputation is the most important aspect of trust in online shopping among the B40 group of consumers.

**Conclusion:** This study aims to provide valuable insights into understanding trust issues among B40 consumers in online shopping. It underscores the importance of website security, seller reputation and punctual delivery in shaping the level of trust.

